# Co-Adjuvant Therapy Efficacy of Catechin and Procyanidin B2 with Docetaxel on Hormone-Related Cancers In Vitro

**DOI:** 10.3390/ijms22137178

**Published:** 2021-07-02

**Authors:** Mª Jesús Núñez-Iglesias, Silvia Novio, Carlota García, Mª Elena Pérez-Muñuzuri, María-Carmen Martínez, José-Luis Santiago, Susana Boso, Pilar Gago, Manuel Freire-Garabal

**Affiliations:** 1 SNL Laboratory, School of Medicine and Dentistry, University of Santiago de Compostela, c/San Francisco, s/n, Santiago de Compostela, 15782 A Coruña, Spain; mjesus.nunez@usc.es (M.J.N.-I.); carlota.garcia.santiago@rai.usc.es (C.G.); mariaelena.perez@usc.es (M.E.P.-M.); manuel.freire-garabal@usc.es (M.F.-G.); 2 Group of Viticulture, Olive and Rose (VIOR), Misión Biológica de Galicia, Consejo Superior de Investigaciones Científicas (CSIC), Carballeira 8, 36143 Salcedo, Spain; carmenmartinez@mbg.csic.es (M.-C.M.); santi@mbg.csic.es (J.-L.S.); susanab@mbg.csic.es (S.B.); pgago@mbg.csic.es (P.G.)

**Keywords:** antitumor activity, catechin, docetaxel, docetaxel-sensitization, hormone-related cancer, natural substances, plant co-adjuvant therapy, procyanidin

## Abstract

Prostate (PC) and breast cancer (BC) are heterogeneous hormonal cancers. Treatment resistance and adverse effects are the main limitations of conventional chemotherapy treatment. The use of sensitizing agents could improve the effectiveness of chemotherapeutic drugs as well as obviate these limitations. This study analyzes the effect of single catechin (CAT), procyanidin B2 (ProB2) treatment as well as the co-adjuvant treatment of each of these compounds with docetaxel (DOCE). We used PC- and BC-derived cell lines (PC3, DU-145, T47D, MCF-7 and MDA-MB-231). The short and long-term pro-apoptotic, anti-proliferative and anti-migratory effects were analyzed. RT-qPCR was used to discover molecular bases of the therapeutic efficacy of these compounds. ProB2 treatment induced a two- to five-fold increase in anti-proliferative and pro-apoptotic effects compared to single DOCE treatment, and also had a more sensitizing effect than DOCE on DU145 cells. Regarding BC cells, ProB2- and CAT-mediated sensitization to DOCE anti-proliferative and pro-apoptotic effects was cell-independent and cell-dependent, respectively. Combined treatment led to high-efficacy effects on MCF-7 cells, which were associated to the up-regulation of CDKN1A, BAX, caspase 9 and E-cadherin mRNA under combined treatment compared to single DOCE treatment. CAT and ProB2 can enhance the efficacy of DOCE therapy on PC and BC cells by the sensitizing mechanism.

## 1. Introduction

Breast and prostate cancers are hormone-related cancers, since their appearance and development is modulated by gonadal steroids. They both present underlying biological similarities [1,2] and are the first and second most common cancer in women and men, respectively [3]. Furthermore, familial history of breast cancer (BC) is associated with prostate cancer (PC) [4] and vice versa [5]. These cancers are highly heterogeneous at cellular and molecular level. Molecular signatures based on gene expression pattern include hormonal receptors such as androgen receptor (AR), estrogen receptor (ER), human epidermal growth factor receptor 2 (HER2) and progesterone receptor (PR). Molecular subtypes can predict baseline prognosis, distant relapse-free survival and therapeutic decision-making strategies [6,7,8,9,10,11,12,13,14].

Docetaxel (DOCE) is a taxane derivate used in BC and PC. Despite its long success, DOCE has a number of problems in use, such as drug-related cumulative toxicity and chemoresistance [15,16,17].

Many dietary phytochemicals are being examined as potential chemopreventive and chemosensitizer agents. The term chemopreventive entails the administration of synthetic, natural or biochemical compounds to prevent the appearance of cancer or even to inhibit or reverse its progression and dissemination [18]. Further, some new therapeutic approaches include the combination of natural compounds with chemotherapeutic drugs to enhance chemotherapy efficacy (chemosensitization) [15] and reduce chemotherapy-induced adverse effects [15,17,18].

Among all dietary phytochemicals advocated for BC and PC therapy, proanthocyanidins have received much attention since they showed potential biological activity in diabetes, cardiovascular diseases, inflammation, infection and cancer [19,20]. In particular, catechins (CATs) seem to be promising chemopreventive agents not only of high-grade prostatic intraepithelial neoplasia (the precursor form of PC) [21,22] but also localized and advanced PC [23].

Proanthocyanidins are compounds that have a flavan-3-ol structure but show wide structural diversity due to their different monomeric constituents, degree of oligomerization and connectivity between the flavan units [24]. The most common flavan-3-ols are catechin (CAT) and epicatechin. The assembly of flavan-3-ol monomer units gives proanthocyanidin oligomers and proanthocyanidin polymers linked mainly through B-type bonds (C4→C8 or C4→C6 bonds). Additionally, they can have another bond between C2→C7 (A-type bond). The proanthocyanidins composed exclusively of CAT and epicatechin are designated as procyanidins (Pro). Dimeric Pro are Pro A1, A2, B1, B2, B3, B4, B5, B6, B8, trimeric Pro are C1, C2, and tetrameric Pro are acatannin A2 and cinnamtannin A2 [25].

CAT and Pro are present in grape seeds and are considered to contribute to the beneficious health effects of wine and grape juice, such as anti-oxidant and anti-carcinogenic effects. CATs’ content depends on, between other factors, geographical origin [26].

The aims of the present research were to examine the in vitro efficacy of ProB2 and CAT compared to the gold standard DOCE, as well as the efficacy of co-adjuvant therapy with CAT and ProB2 plus DOCE on hormone-related BC and PC cancer. Taking into account that BC [8,9,10,27] and PC [11,12,13,14] have different molecular subtypes, we used human tumor-derived cell lines that mirror many molecular and clinical aspects of BC and PC in the clinical practice and can play important roles in translational medicine and biology [28]. We observed the cell line-specific efficacy of single and combined treatments as well as the cell line-specific efficacy of CAT and ProB2 sensitizing effects.

## 2. Results

### 2.1. Cytotoxic Effects on Health Cells

As chemotherapy treatments may cause damage to both cancerous and healthy cells, we investigated the cytotoxicity of ProB2 and CAT on healthy cells at first. The selected doses used on cancer cells were chosen according to the toxicity levels of ProB2 and CAT treatment on mammary and prostate healthy cells.

**Figure 1:**
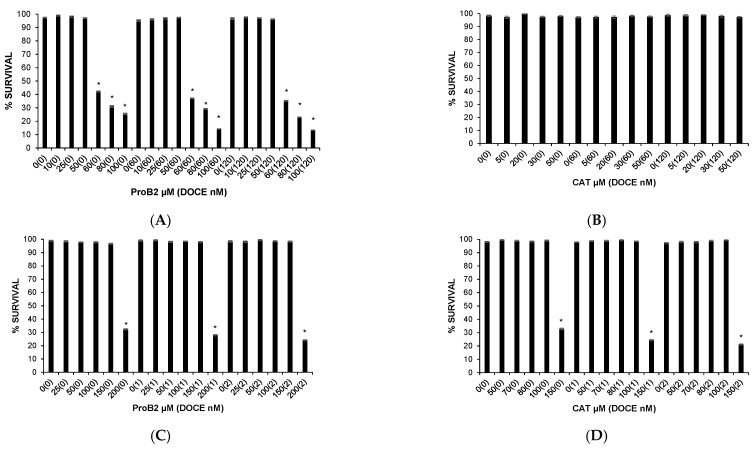
Effect of single or combined therapy on proliferation of healthy cells (trypan blue dye exclusion assay). (**A**) Effect of different concentrations of ProB2 (10–100 μM), with and without DOCE (60–120 nM) on healthy mammary cells. (**B**) Effect of different concentrations of CAT (5–50 μM), with and without DOCE (60–120 nM) on healthy mammary cells. (**C**) Effect of different concentrations of ProB2 (25–200 μM), with and without DOCE (1–2 nM) on healthy prostate cells. (**D**) Effect of different concentrations of CAT (50–150 μM), with and without DOCE (1–2 nM) on healthy prostate cells. The data are means ± SD (*n* = 3). * *p* < 0.05, significantly different compared with control treatment. Abbreviations: CAT, catechin; DOCE, docetaxel; ProB2, procyanidin B2.

The survival of mammary and prostate healthy cells was minimally affected (differences *p* > 0.05) by ProB2 and CAT. Thus, 97.31% ± 0.76 and 98.04% ± 0.78 of mammary health cells (*p* > 0.05 compared with controls) were viable after 72 h treatment with 50 µM ProB2 (Figure 1A, Table 1B) and 50 µM CAT (Figure 1B, Table 1B), respectively. Further, 96.62% ± 0.78 and 99.05% ± 0.68 of prostate health cells were viable following a 72 h exposure to 150 µM ProB2 (Figure 1C, Table 1A) and 100 µM CAT (Figure 1D, Table 1A), respectively.

**Figure 2:**
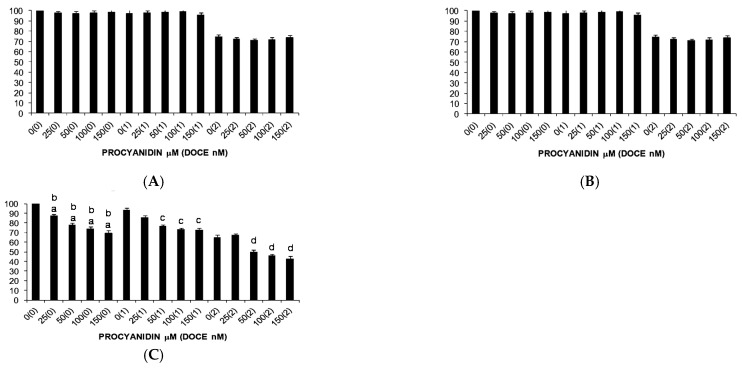
Trypan blue dye exclusion assay results showing viability of DU 145 PC cells under different concentrations of the test compound (ProB2, DOCE, ProB2 + DOCE) at 24 h (**A**), 48 h (**B**) and 72 h (**C**). Columns, mean; bars, SD (*n* = 3). ^a^ represents the significant difference between ProB2 treatment alone for 72 h and control treatment at 24 h; ^b^ represents the significant difference between ProB2 treatment alone for 72 h and control treatment at 48 h; ^c^ represents the significant difference between ProB2 treatment alone for 72 h and 1 nM DOCE treatment alone; ^d^ represents the significant difference between ProB2 treatment alone for 72 h and 2 nM DOCE treatment alone. Abbreviations: CAT, catechin; DOCE, docetaxel; ProB2, procyanidin B2.

**Figure 3:**
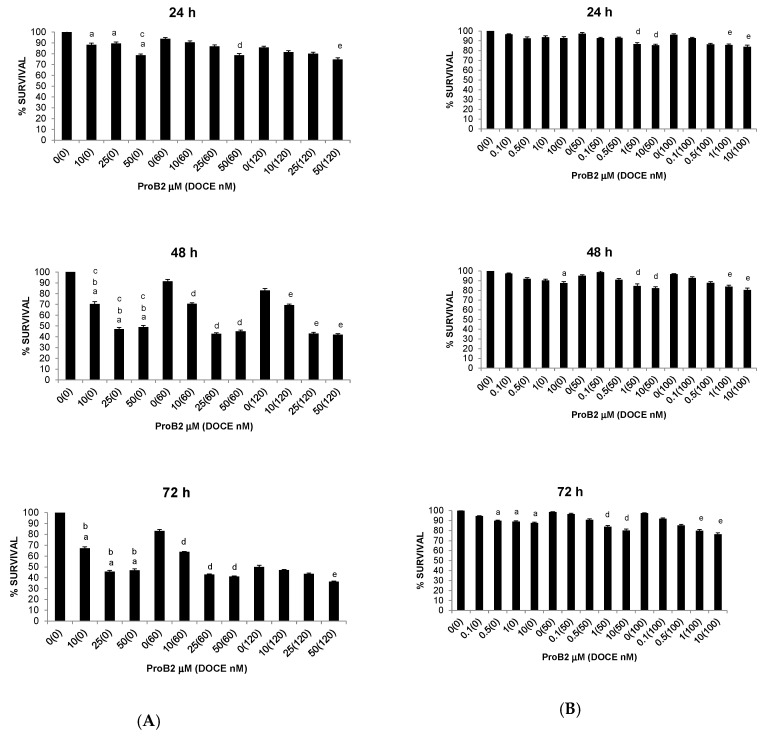
Trypan blue dye exclusion assay results showing viability of MCF-7 (**A**) and T47D (**B**) BC cells under different concentrations of the test compound (ProB2, DOCE, ProB2 + DOCE) at 24 h, 48 h and 72 h. Columns, mean; bars, SE (*n* = 3). ^a^ represents the significant difference between ProB2 treatment alone and control treatment; ^b^ represents the significant difference between ProB2 treatment alone for 48 or 72 h and ProB2 treatment alone for 24 h; ^c^ represents the significant difference between ProB2 treatment alone and 120 nM DOCE alone; ^d^ represents the significant difference between combination treatment (ProB2 + DOCE 60 nM or DOCE 50 nM) and 60 nM DOCE or 50 nM DOCE alone; ^e^ represents the significant difference between combination treatment (ProB2 + DOCE 120 Nm or DOCE 50 nM) and 120 nM DOCE or DOCE 50 nM alone (*p* < 0.05; one-way ANOVA). Abbreviations: DOCE, docetaxel; ProB2, procyanidin B2.

It should be noted that the same treatment regimen (dose and treatment time) significantly reduced the viability of tumor cells. By way of illustration, the survival rate was ≈70% and 48% in DU145 PC cells (Figure 2) and MCF-7 BC cells (Figure 3A), respectively, after 72 h exposure to 150 µM ProB2.

### 2.2. Antitumor Effects

#### 2.2.1. Anti-Proliferative Activity

Time course cytotoxic effects were evaluated at different time points after exposure to single ProB2, CAT and DOCE treatment or ProB2 plus DOCE, CAT plus DOCE co-adjuvant therapy in comparison to untreated cells and single DOCE treatment. We used PC as well as BC lines with different degrees of malignancy and hormonal receptor status.

Single ProB2 treatment induced dose- and time-dependent cytotoxicity on DU145 PC cells (Figure 2), whereas single CAT treatment did not induce a significant cell death toward the PC cell lines, and over the concentration ranges tested.

**Figure 4:**
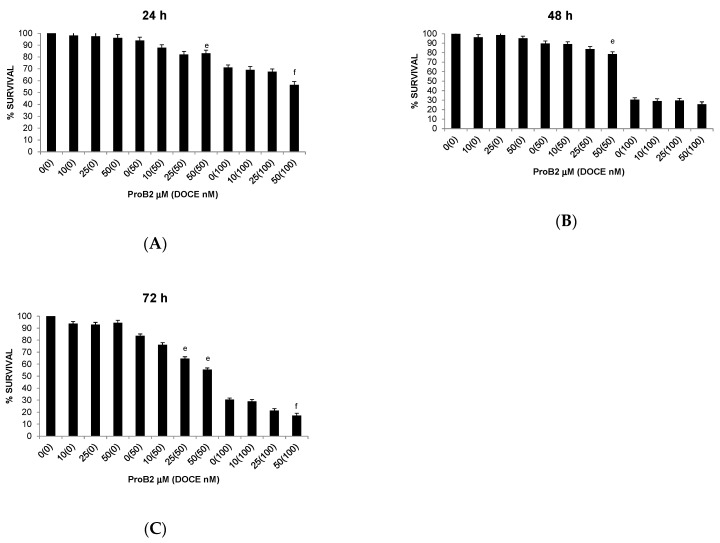
Trypan blue dye exclusion assay results showing viability of MDA-MB-231 cells under different concentrations of the test compound (ProB2, DOCE, ProB2 + DOCE) at 24 h (**A**), 48 h (**B**) and 72 h (**C**). Columns, mean; bars, SE (*n* = 3). ^e^ represents the significant difference between combination treatment (ProB2 + DOCE 50 nM) and 50 nM DOCE alone; ^f^ represents the significant difference between combination treatment (ProB2 + DOCE 100 nM) and 100 nM DOCE alone (*p* < 0.05; one-way ANOVA). Abbreviations: DOCE, docetaxel; ProB2, procyanidin B2.

The effect of single ProB2 treatment on proliferation of T47D, MCF-7 and MDA-MB-231 cells is shown in Figure 3 and Figure 4. ProB2 significantly reduced the viability of MCF-7 (Figure 3A) and T47D (Figure 3B) cells (*p* < 0.05); however, the viability of MDA-MB-231 cells was unaffected (Figure 4). A comparison of both cell lines revealed that ProB2 had anti-proliferative effect in a dose- and time-dependent manner on MCF-7 cells, and only in a dose-dependent manner on T47D cells. Despite the fact that the effect was more pronounced in MCF-7 cells (73% at 24 h exposure to 50 µM), the inhibition of the growth of T47D ones was observed with lower doses (10–50 µM for MCF-7 versus 0.5–10 µM for T47D). Concretely, the maximum inhibitory effect on MCF-7 cells (47% at 72 h) was observed with the highest dose tested that was not toxic to normal mammary cells, whereas the maximum effectiveness on T47D cells (87% at 72 h) was noted at 10 µM.

Moreover, ProB2 was more effective on MCF-7 cells than DOCE time-course of treatment (Figure 3A). A comparison of the two treatments reveals that ProB2 (50 µM) reduced cell proliferative activity by ≈21% and 51%, whereas that of 120 nM DOCE cell proliferative activity by ≈14% and 17% on exposure for 24 vs. 48 h, respectively. This effect was not observed in the other two BC cell lines.

**Figure 5:**
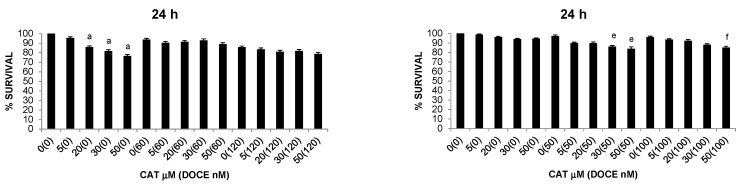

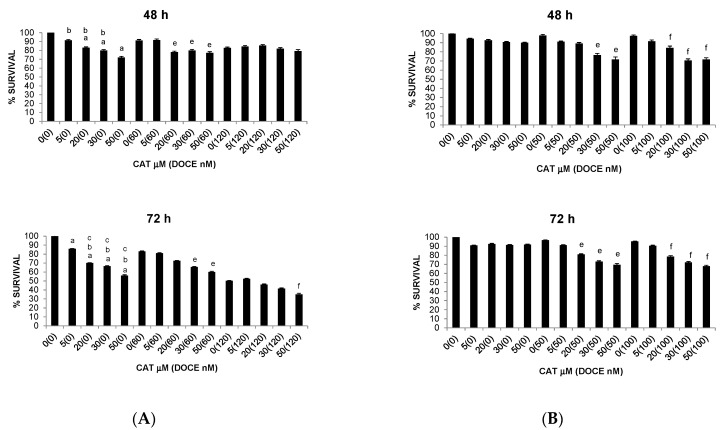
Trypan blue dye exclusion assay results showing viability of MCF-7 (**A**) and T47D (**B**) BC cells under different concentrations of the test compound (CAT, DOCE, CAT + DOCE) at 24 h, 48 h and 72 h. Columns, mean; bars, SE (*n* = 3). ^a^ represents the significant difference between CAT treatment alone and control treatment; ^b^ represents the significant difference between CAT treatment alone for 48 or 72 h and CAT treatment alone for 24 h; ^c^ represents the significant difference between CAT treatment alone for 72 h and CAT treatment alone for 48 h; ^e^ represents the significant difference between combination treatment (ProB2 + DOCE 60 Nm or DOCE 50 nM) and 60 nM DOCE or DOCE 50 nM alone; ^f^ represents the significant difference between combination treatment (ProB2 + DOCE 120 nM or DOCE 100 nM) and 120 nM DOCE or DOCE 100 nM alone (*p* < 0.05; one-way ANOVA). Abbreviations: CAT, catechin; DOCE, docetaxel.

**Figure 6:**
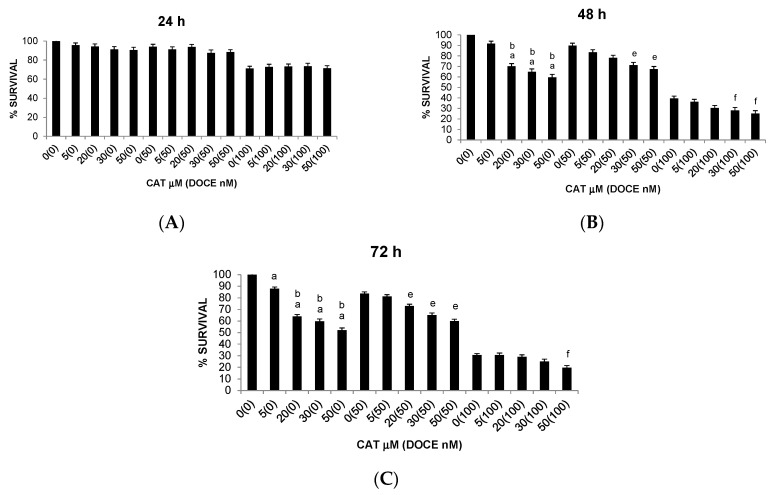
Trypan blue dye exclusion assay results showing viability of MDA-MB-231 cells under different concentrations of the test compound (CAT, DOCE, CAT + DOCE) at 24 h (**A**), 48 h (**B**) and 72 h (**C**). Columns, mean; bars, SE (*n* = 3). ^a^ represents the significant difference between CAT treatment alone and control treatment; ^b^ represents the significant difference between CAT treatment alone for 48 or 72 h and CAT treatment alone for 24 h; ^e^ represents the significant difference between combination treatment (ProB2 + DOCE 50 nM) and DOCE 50 nM alone; ^f^ represents the significant difference between combination treatment (ProB2 + DOCE 100 nM) and DOCE 100 nM alone (*p* < 0.05; one-way ANOVA). Abbreviations: CAT, catechin; DOCE, docetaxel.

As shown in Figure 5 and Figure 6, CAT significantly inhibited the proliferative activity of MCF-7 and MDA-MB-231 cells, which was time- and dose-dependent. Comparing the cell lines, it can be seen that the effect began earlier in MCF-7 (24 h) cells but was more pronounced in MDA-MB-231 cells (Figure 5A and Figure 6). By way of illustration, the maximum inhibitory effect (concentration of 50 µM) was 28% vs. 40% (48 h) and 44% vs. 48% (72 h) in MCF-7 and MDA-MB-231 cells, respectively. In contrast, CAT showed no significant effect on the growth rate of T47D cells. The most striking result to emerge from the data is that CAT was more effective than the chemotherapy drug (DOCE) at inhibiting the proliferation of MCF-7 (Figure 5A) and MDA-MB-231 cells (Figure 6).

#### 2.2.2. Pro-Apoptotic Activity

With the anti-proliferative effect of ProB2 and CAT established, we sought to characterize whether the effects of these compounds were due to apoptosis.

**Figure 7:**
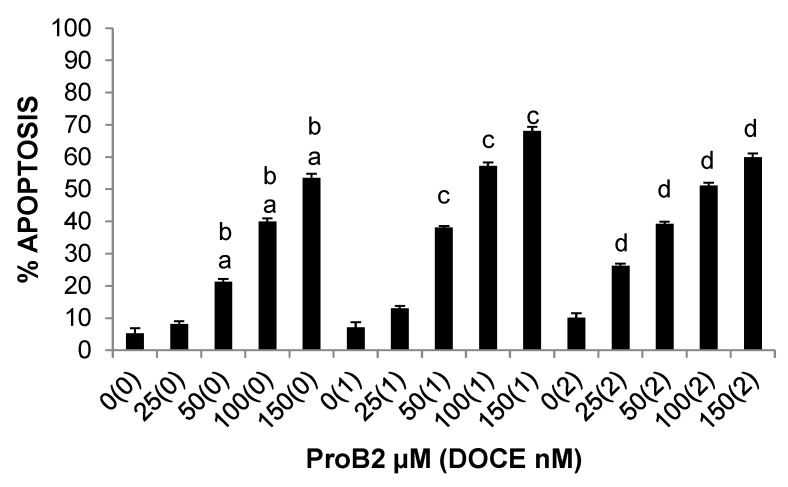
DAPI assay results showing apoptosis of DU145 cells under different concentrations of the test compound (ProB2, DOCE, ProB2 + DOCE) at 72 h. Columns, mean; bars, SE (*n* = 3). ^a^ represents the significant difference between ProB2 treatment alone and control treatment; ^b^ represents the significant difference between ProB2 treatment alone and 2 nM DOCE alone; ^c^ represents the significant difference between combination treatment (ProB2 + DOCE 1 nM) and DOCE 1 nM alone; ^d^ represents the significant difference between combination treatment (ProB2 + DOCE 2 nM) and DOCE 2 nM alone (*p* < 0.05; one-way ANOVA). Abbreviations: DOCE, docetaxel; ProB2, procyanidin B2.

Figure 7 provides the results obtained from the analysis of apoptosis in DU145 cells. This figure is quite revealing in several ways. First, it shows a dose-dependent increase in the induction of apoptosis under ProB2 treatment, compared to untreated control cells. Second, there is a clear pro-apoptotic activity of ProB2-treated DU145 cells, which exhibited a two- to five-fold increase compared to DOCE-treated cells.

**Figure 8:**
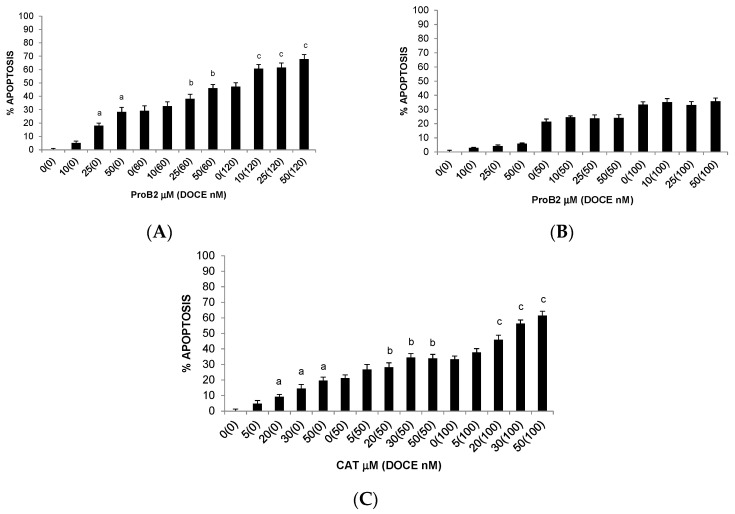
DAPI assay results showing apoptosis of MCF-7 (**A**), T47D (**B**) and MDA-MB-231 (**C**) cells under different concentrations of the test compound (CAT, DOCE, CAT + DOCE) at 72 h. Columns, mean; bars, SE (*n* = 3). ^a^ represents the significant difference between CAT treatment alone and control treatment; ^b^ represents the significant difference between combination treatment (CAT + DOCE 50 nM or DOCE 60 nM) and low-dose DOCE alone; ^c^ represents the significant difference between combination treatment (CAT + DOCE 100 nM or DOCE 120 nM) and high-dose DOCE alone (*p* < 0.05; one-way ANOVA). Abbreviations: CAT, catechin; DOCE, docetaxel.

Remarkably, comparing the results between the different BC cell lines, it can be seen that ProB2-mediated death of MCF-7 (Figure 8A) and T47D (Figure 8B) cells can be explained by apoptotic mechanisms following a 72 h treatment, but not in MDA-MB-231 cells (Figure 8C). This effect is clearly dose-dependent on MCF-7 cells. On the other hand, the apoptotic rates of MCF-7, MDA-MB-231 and T47D cells treated with DOCE were of up to 47%, 33% and 19%, respectively.

From Figure 8, we can see a dose-dependent increase in apoptosis when MCF-7 (Figure 8A) and MDA-MB-231 (Figure 8C) cell lines were treated with CAT in relation to untreated control cells, with maximal effects for both cells being at 50 μM. By comparison, CAT had very little effect on the apoptosis of T47D cells relative to control (Figure 8B).

In summary, these results show that only MCF-7 cells, when exposed to ProB2 or CAT, exhibited a concentration-dependent increase in apoptosis. The results suggest the feasibility of cancer cell type-mediated effect. The cells used show different genetic background regarding hormonal and p53 status as well as tumor type. MDA-MB-231 cells are TNBC cells (ER-, PR -, HERB2-), AR- and p53 mutant [29,30,31,32], whereas both MCF-7 and T47D cells are ER+, PR +, HERB2-, AR+ but p53 wild type (wtp53) [30,31,32] and p53 mutant (mtp53) [29,30,32], respectively. On the other hand, MDA-MB-231 cells are claudin low [28,33], basal B adenocarcinoma cells in contrast to MCF-7 and T47D cells. They both are luminal [28,34,35,36] but A- and B-type invasive ductal carcinoma cells, respectively [37].

#### 2.2.3. Sensitization of Cancer Cells to DOCE Growth-Suppressive Effect

In relation to PC cells, ProB2 sensitized DU145 to chemotherapy treatment with DOCE (Figure 2A). Interestingly, when cells were under co-treatment with ProB2 plus DOCE, ProB2 increased the anti-proliferative and anti-apoptotic efficacy of DOCE. Compared with 1nM DOCE treatment, ProB2 plus DOCE was from three-fold (50 μg/mL ProB2 plus 1 nM DOCE) up to six-fold (150 μg/mL ProB2 plus 1 Nm DOCE) more effective in inducing both cell growth inhibition and apoptosis after 72 h of treatment. Similar results were observed when compared to single 2 nM DOCE-treated cells with ProB2 plus 2 nM DOCE-treated ones (Figure 3).

Regarding BC cells, co-treatment with ProB2 plus DOCE showed a marked decrease in proliferative activity compared with those treated with a single DOCE treatment (Figure 4 and Figure 5). This effect was observed in all the BC cells tested. For example, the proliferative activity of MDA-MB-231 cells was mildly affected (≈16% decrease) by 60 nM DOCE alone for 72 h, and exposure to co-treatment with 50 μM ProB2 plus 60 nM DOCE decreased proliferative activity ≈45%. Analyzing results according to Lee et al. [38], the combination index was <1 (combination index of 0.04).

Similarly, as shown in Figure 5, the percentages of cell growth inhibition induced by CAT in combination with DOCE were greater than each single agent. CAT sensitized MCF-7, MDA-MB-231 and T47D cells to DOCE treatment. The CAT and DOCE combination significantly inhibited more proliferation of the three BC cell lines than single CAT or single DOCE treatment. For example, the proliferative activity of T47D cells remained almost unchanged after 50 nM DOCE (≈98%) or 50 nM CAT (≈90%) treatment for 72 h. On the contrary, the number of cells was significantly reduced by ≈28% by a 72 h co-treatment with 50 μM CAT plus 50 nM DOCE. The combination index was <1 (combination index 0.03). In relation to the sensitizing effect of ProB2 on apoptosis induced by DOCE, a comparison between single DOCE treatment and ProB2 plus DOCE co-treatment revealed that combination therapy increased the pro-apoptotic efficacy of the chemotherapeutic drug in MCF-7 and T47D but not in MDA-MB-231 cells.

With respect to combination therapy with DOCE plus CAT, it increased the efficacy of the chemotherapeutic drug in MCF-7 and MDA-MB-231. As shown in Figure 8A,C, when MCF-7 and MDA-MB-231 cell lines were treated with higher doses of CAT plus DOCE, they exhibited a stronger percentage of apoptotic cell death than DOCE-treated cells.

Together, these results provide interesting insights into an effective adjunct therapy to enhance DOCE antitumor efficacy.

#### 2.2.4. Colony Formation Test

The first set of analyses showed above examined the impact of ProB2 and CAT, with or without DOCE, on the proliferative activity of different BC and PC cell lines at early time points (24, 48 and 72 h). Further analysis to determine whether the treatments had a persistent (14 days) inhibitory effect on the proliferative activity of DU145 and MCF-7 cells was carried out by a colony formation test.

This test determines the number of cancer cells which maintain their proliferative capacity as well as being able to undergo at least five to six divisions to form a colony after cytotoxic agent treatments or radiotherapy [39].

**Figure 9:**
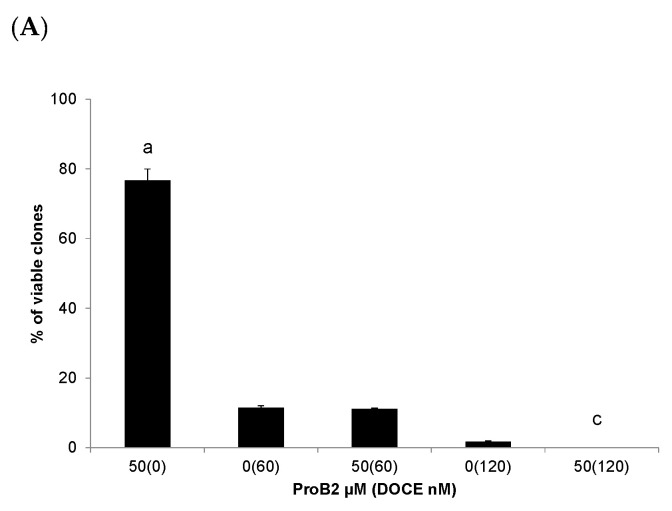

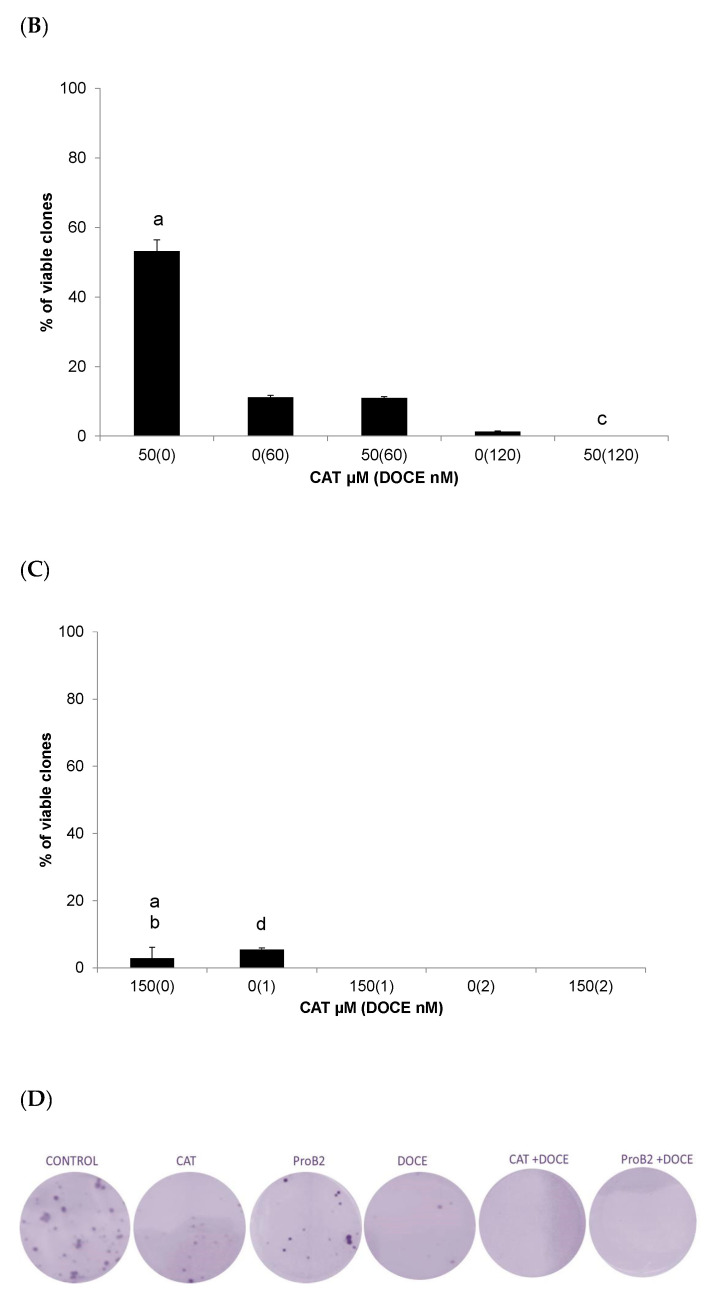
Effect of the treatments for 14 days on the clonogenic activity MCF-7 cells and DU145 cells. Clonogenic assay of MCF-7 cells treated with: (**A**) ProB2, DOCE or ProB2/DOCE combination or (**B**) CAT, DOCE or CAT/DOCE combination. (**C**) Clonogenic assay of DU145 cells treated with CAT, DOCE or CAT/DOCE combination. (**D**) Representative images depicting colony formation for MCF-7 cells treated with 50 µM CAT, 50 µM ProB2, 120 nM DOCE, 50 µM CAT+120 nM DOCE or 50 µM ProB2+120 nM DOCE. Clonogenic assay was performed as described in material and methods. Columns, mean; bars, SE (*n* = 3). ^a^ represents the significant difference between CAT or ProB2 treatment alone and control treatment; ^b^ represents the significant difference between CAT or ProB2 treatment alone and low-dose DOCE; ^c^ represents the significant difference between combination treatment (CAT + DOCE) and DOCE alone; ^d^ represents the significant difference between combination treatment (ProB2 + DOCE) and DOCE alone (*p* < 0.05; one-way ANOVA). Abbreviations: CAT, catechin; DOCE, docetaxel; ProB2, procyanidin B2.

As can be seen from Figure 9C, single treatment with 150 µM ProB2 in DU145 PC cells significantly (*p* < 0.05) reduced clonogenic potential compared to the control, 1 nM DOCE or 2nM DOCE treatments. It is noteworthy that co-treatment with ProB2 plus DOCE drastically blocked the clonogenic potential of DU145 cancer cells, and no colonies were observed.

Single CAT as well as ProB2 treatments in MCF-7 BC cells significantly (*p* < 0.05) reduced clonogenic potential compared to the control (Figure 9A,B). Interestingly, we observed that exposure at higher doses of DOCE (120 nM) combined with CAT or with ProB2 caused a significant inhibition of the clonogenic potential of these BC cells with completely inhibited colony formation.

#### 2.2.5. Cell Migration Assay

Since it is postulated that the study of anti-tumor molecules requires dichotomization into two strategies, anti-proliferative and anti-migratory effects [40], we assessed whether ProB2 (DU145) as well as CAT and ProB2 (MCF-7) could affect the ability of tumor cells to migrate using a transwell assay.

**Figure 10:**
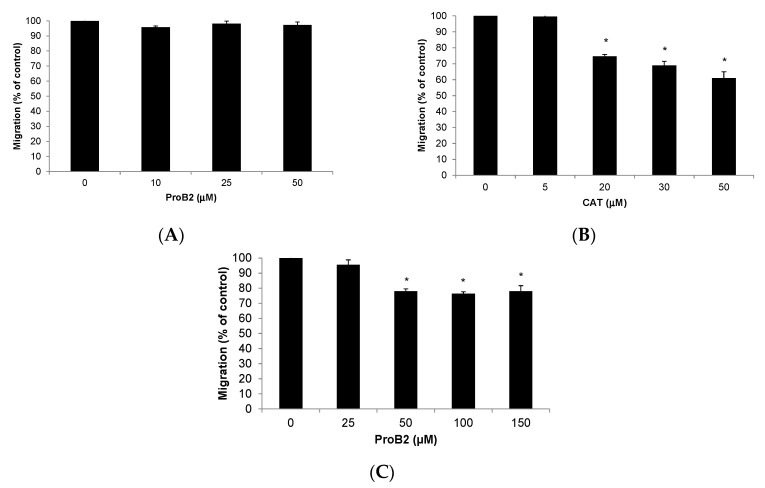
Effects of ProB2 and CAT on cancer cell migration. Cell migration of MCF-7 cells treated with: (**A**) ProB2 or (**B**) CAT. (**C**) Cell migration of DU145 cells treated with ProB2. Columns, mean; bars, SE (*n* = 3). * represents the significant difference between ProB2 or CAT treatment and control treatment (*p* < 0.05; one-way ANOVA). Abbreviations: CAT, catechin; DOCE, docetaxel; ProB2, procyanidin B2.

ProB2 treatment showed higher anti-migratory effect than the control treatment on DU145 cells (Figure 10C). On the other hand, the migration ability of MCF-7 cells exposed to CAT treatment was significantly decreased, compared with untreated control cells (*p* < 0.05). The maximum effect appeared at 50 µM (Figure 10B). At the highest concentration of CAT tested (50 µg/mL), the percent migration decreased to 40% (Figure 10B). However, there was no significant change in MCF cells’ migration after treatment (10–50 µM) of ProB2 (Figure 10A).

#### 2.2.6. Gene expression

As stated previously, CAT and ProB2 compounds were found to be highly effective on MCF-7 cells in comparison to the other cell lines. Furthermore, these compounds sensitized MCF-7 cells to DOCE anti-proliferative and anti-apoptotic effects, as well as reduced migration. In order to identify the main genes and pathways implicated in these effects, a gene expression profiling analysis was performed by RT-qPCR (reverse transcription–quantitative polymerase chain reaction).

- Sensitization of cancer cells to DOCE growth-suppressive effect

**Figure 11:**
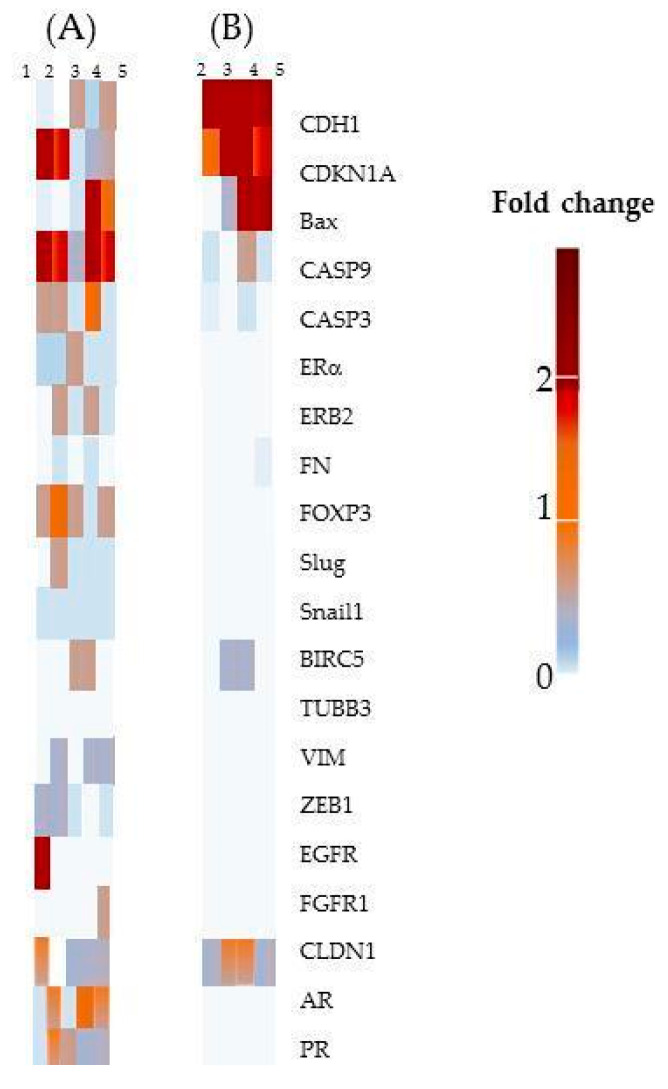
Fold change in gene mRNA expression of the 20-gene signature in MCF-7 cells. (**A**) Fold change in comparison to control. (**B**) Fold of change in comparison to DOCE. 1, DOCE (120 nM)-treated cells; 2, CAT (50 µM)-treated cells; 3, ProB2 (50 µM)-treated cells; 4, CAT (50 µM) plus DOCE (120 nM)-treated cells; 5, ProB2 (50 µM) plus DOCE (120 nM)-treated cells. Abbreviations: AR, androgenic receptor; BAX, BCL-2-associated X protein; BIRC5, baculoviral inhibitor of apoptosis repeat-containing 5; CDH1, cadherin type 1, E-cadherin; CDKN1A, cyclin-dependent kinase inhibitor 1A; CASP9, caspase-9; CASP3, caspase-3; CAT, catechin; CLDN1, claudin 1; DOCE, docetaxel; EGFR, epidermal growth factor receptor; FGFR1, fibroblast growth factor receptor 1; FN; fibronectin; FOXP3, forkhead box protein P3; ProB2, procyanidin B2; SLUG, snail family zinc finger 2; ZEB1, zinc finger E-box-binding homeobox 1; VIM, vimentin.

Different genes involved in the mechanism of proliferative activity, apoptosis, and/or taxane chemosensitization/chemoresistance [41,42,43,44,45,46,47,48,49,50] were analyzed (Figure 11). We observed that combined therapy with ProB2 plus DOCE or CAT plus DOCE up-regulated CDKN1A (cyclin-dependent kinase inhibitor 1A), BAX (BCL-2-associated X protein), CASP9 (caspase-9) and E-cadherine. What is striking in Table 2 is that CDKN1A and BAX mRNA levels increased by almost eight-fold in CAT plus DOCE-treated cells compared to single DOCE treatment. Additionally, CDKN1A and Bax mRNA levels showed a ≈ two-fold increase in ProB2 plus DOCE-treated cells in relation to DOCE-treated ones. Furthermore, as indicated below, the single treatment of CAT (67-fold) or ProB2 (13-fold) as well as in combination with DOCE (39-fold CAT plus DOCE vs. 9-fold ProB2 plus DOCE) greatly increased the expression of E-cadherin, in relation to DOCE. It is already known that CDKN1A [41,42], BAX [43,44,45], CASP9 [46,47] and E-cadherine are involved in tumor growth suppression and apoptosis [48]. By contrast, E-cadherine suppression is associated to DOCE chemoresistance in hormonal tumors [49,50].

In our study, there was no evident effect on caspase-3 (CASP3) mRNA levels after single or combined treatments. It is known that MCF7 is a cell line with the deletion of the CASP3-encoding gene [46,47]. Concerning CASP9, up-regulated expression was observed after CAT plus DOCE combined treatment (eight-fold increase) but lower compared to the DOCE single one (18-fold increase) (Table 2). CASP3 and CASP9 are involved in taxane-induced cell death [46].

Overall, our results suggest that CDKN1A, BAX, CASP9 and E-cadherin up-regulated expression contributed to the ProB2- and CAT-induced sensitization to DOCE in MCF-7 cells.

- CAT and ProB2-induced anti-migratory effect

To identify the mechanisms which might account for the observed decrease in cell migration, the expression of epithelial–mesenchymal transition (EMT)-related genes [48,49,50,51] was studied. As Table 2 shows, E-cadherin (epithelial phenotype-associated gene) mRNA gene expression was about 67-fold and 13-fold up-regulated in CAT and ProB2-treated cells, respectively, compared to DOCE treatment. It is noteworthy that E-cadherin expression was increased in CAT plus DOCE (39-fold increase) and ProB2 plus DOCE (nine-fold increase)-treated cells compared to single DOCE-treated cells (*p* < 0.05). These results suggest that E-cadherin up-regulation by CAT or PRoB2 efficiently sensitizes MCF-7 cells to DOCE. After single CAT, ProB2 treatments and CAT or PRoB2 combined with DOCE, there was no significant change observed in mRNA gene expression in ZEB1 (zinc finger E-box-binding homeobox 1), FN (fibronectin), SLUG (snail family zinc finger 2), VIM (vimentin), CLDN1 (claudin 1), EGFR (epidermal growth factor receptor), FGFR1 (fibroblast growth factor receptor 1), and FOXOP3 (Figure 11). All of them are mesenchymal phenotype-associated transcription factors which are associated to migration in BC [52] and DOCE chemoresistance in MCF-7 cells [53] through the repression of epithelial marker E-cadherin.

## 3. Discussion

In clinical practice, the expression of hormonal receptors determines the response to treatment, prognosis and survival. As an example, according to the American Society of Clinical Oncology (ASCO) [56] and European Society for Medical Oncology (ESMO) [57] guidelines, the analysis of the hormonal receptor characteristics constitutes a standard in BC and PC in order to apply therapy decisions. The pattern of expression of receptors determines treatment that includes hormonal treatment, chemotherapy, antibodies or a combination of these. BC and PC cells that express the ER (ER+), PR (PR+), AR (AR+) depend on estrogens, progesterone and androgens for their growth and are more responsive to endocrine ablation.

As can be seen from Table 3, we used BC and PC cell lines that reflect the genomic diversity as well as tumor type and aggressiveness [34,58]. The BC cells used are considered to possess similarity to BC tumors, such as PAM50 (Prediction Analysis for Microarrays) and whole gene expression profile [34]. DU145 and PC3 are considered as metastatic castrate-resistant prostate cancer (mCRPC). CRPC involves cancer cells becoming refractory to treatment and stopping responding to androgen deprivation therapy. Androgen deprivation therapy aims to block the effect of androgens in CRPC, as they have a proliferative effect. In clinical practice, between 10% and 50% of PC progresses to mCRPC within 3 years of diagnosis and, despite advances in treatment, remains lethal. When CRPC occurs, new treatments are required to reduce the androgenic signal and consequently tumor growth, although resistance is usually developed as well [59].

The first question in this study sought to determine the in vitro efficacy of ProB2 and CAT on hormone-related cancer cell lines compared to the gold standard DOCE. In the present study, we have verified that the treatment regimen (dose and treatment period) of ProB2 and CAT with or without DOCE has high antitumor effects without evident toxicity on healthy breast and prostate cells. DOCE is a chemotherapeutic agent that acts on the microtubules of cancer cells inducing cell cycle arrest and apoptosis. Its clinical benefits are limited both by its cumulative toxicity and by responsiveness or resistance to it [60]. Hence, there is growing interest in the search for compounds that show an effect similar to DOCE or sensitize tumor cells to this drug with minimal effect on healthy cells. Recent studies analyzed the chemosensitization of cancer cells by natural compounds, although most of them did not show the effect of therapeutic doses on healthy cells [61,62,63,64,65]. In this study, doses of ProB, CAT, ProB2 plus DOCE or CAT plus DOCE, with minimal impact on the viability of healthy breast or prostate cells (viability ranging from 96 to 98%), drastically decreased viability in PC and BC cancer cells (viability between 73% and 45%, depending on the compound and cell type). Future research questions that could be asked include the effects on other healthy cells such as blood cells, human reproductive cells, etc. Our ongoing research shows that CAT and ProB2 did not exhibit evident toxic effects on human fibroblast (data not showed). This is the cell most common in connective tissue, widely distributed throughout the human body.

One of the most interesting findings of this study was a cell line-specific efficacy of single ProB2 and CAT treatments. With respect to ProB2, it showed a higher anti-proliferative effect than DOCE on DU145 cells, at all doses tested at 72 h. In contrast, it did not show effects on PC3 PC cells. Additionally, ProB2 exhibited more anti-proliferative effects than DOCE on MCF-7 BC cells, at all doses tested and through the treatment period. This effect was not observed in T47D and MDA-MB-231 cells. CAT exhibited more effective anti-proliferative effects than DOCE on MCF-7 and MDA-MB-231 cells but not on T47D breast cancer cells.

**Figure 12:**
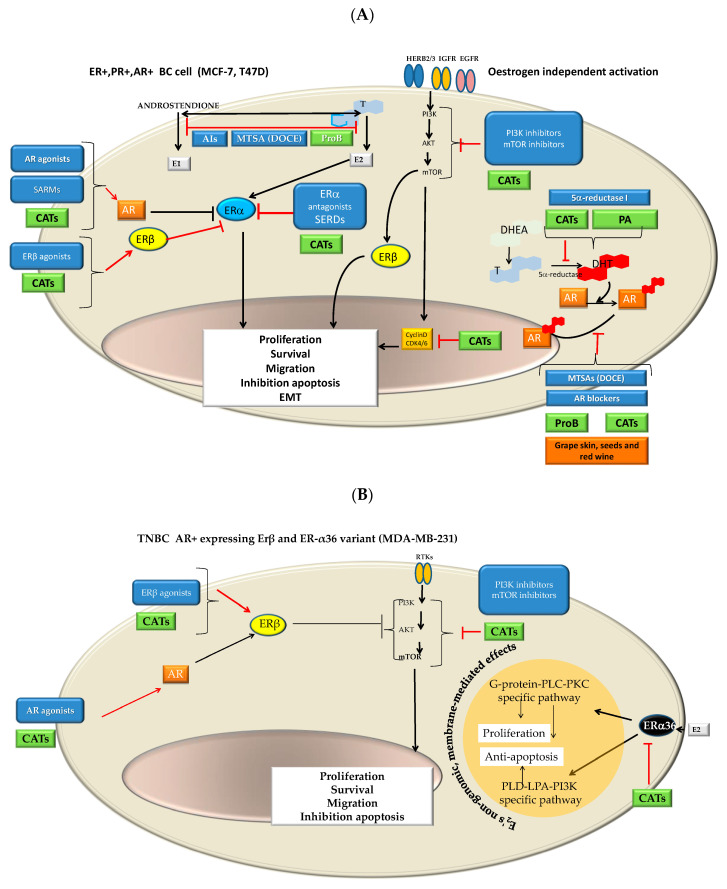

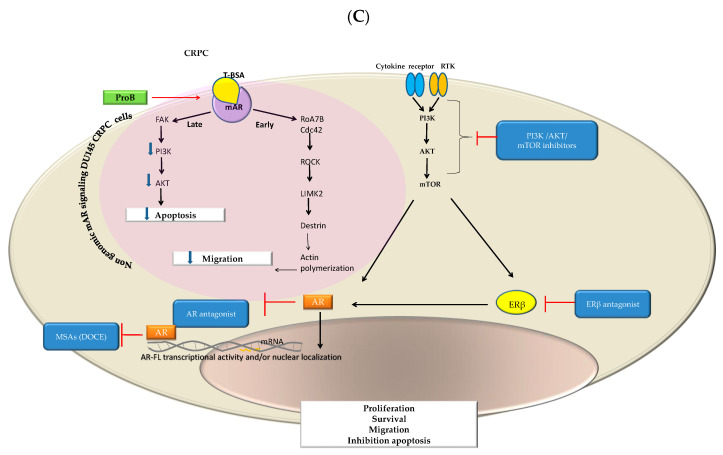
Schematic representation of proposed mechanism by which CAT and ProB2 with and without DOCE could promote anti-tumor effects. Panels (**A**–**C**) show therapeutic targets in BC and PC with the corresponding drugs used in clinical practice (boxes in blue). Likewise, the targets on which CATs and/or ProB (green boxes) can act are observed. Black truncated arrows and lines indicate mechanisms that stimulate tumor growth and invasion. Red truncated arrows/lines show the effects of drugs as well as CAT/ProB. Therapeutic targets depending on the type of BC and PC are based on Karamouzis et al. [119] with modifications. It is observed how the different targets and mechanisms vary depending on the cellular characteristics. Based on this aspect, different cell sensitivity can be expected under single (CAT, ProB, DOCE treatment) or co-adjuvant treatment (CAT plus DOCE or ProB2 plus DOCE). Panel (**A**) represents ER+, PR+, AR+ BC (MCF-7 and T47D cells) while panel (**B**) represents TNBC BC (MDA-MB-231 cells). Orange circle represents E2’s non-genomic, membrane-mediated effects according to Chaudhri et al. [120]. Panel (**C**) represents CRPC (DU-145 and PC3 cell lines). The circle in purple color corresponds to the schematic representation of non-genomic mAR signaling in DU145 CRPC based on Papadopoulou et al. [121]. Meanwhile, BC and PC are hormone cancers; different hormones such as estrogens, progesterone and androgens are involved in their growth and metastatization. The pro-tumor effect of these hormones is largely established through their binding to their corresponding receptors. Therefore, the therapy of these cancers includes molecular target therapies such as selective modulators of hormone receptors as well as selective inhibitors of enzymes related to the synthesis of steroid hormones [75,122]. Namely, selective ER modulators (SERMs), selective ER down-regulators (SERDs), selective PR modulators (SPRMs), selective androgen receptors modulators (SARMs), aromatase inhibitors (Ais) and 5α-reductase inhibitors, represented in panels A, B and C. SERMs, by binding to the ER, prevent binding of estrogens to that receptor and thereby estrogen-stimulated cell proliferation, whereas SERDs down-regulate the ER. SAMRs interact with AR, preventing tumor growth and metastatization. AIs inhibit the synthesis of estrogens (aromatase enzyme is involved in the conversion of the testosterone to estradiol). 5α-reductase converts testosterone into a more active androgen called 5α-DTH and also metabolizes progesterone to 5αDHP, being an important regulator of androgens and progesterone effects. Testosterone and 5α-DTH bind to AR and induce prostate hypertrophy as well as PC. 5α-reductase inhibitors block 5α-DTH production. CATs, by acting on hormonal receptors, could have pharmacological actions similar to those of modulators of these receptors (SERMs, SERDs, SPRMs, SARMs) [66,67,68,69,70,71,72,73,74,75,82]. In addition, they would act by inhibiting aromatase [76,77,78,79] or 5-α reductase [81]. DOCE causes AI in luminal BC cells and alters AR transcriptional activity and/or nuclear localization (panel A). Furthermore, the belief that DOCE acts by interfering with the mechanism of action of AR is reinforced. DOCE could prevent AR translocation into the nucleus. Indeed, there is correlation between clinical response and AR cytoplasmic retention in circulating tumor cells isolated from mCRPC treated with DOCE [123]. Abbreviations: AIs, aromatase inhibitors; Akt, protein kinase B; AR, androgen receptor; BSA, bovine serum albumin; CAT, catechin; CRPC, castrate resistant prostate cancer; DHEA, dehydroepiandrosterone; DHP, dihydroprogesterone;DHT, dihydrotestosterone; DOCE, docetaxel; ERα, estrogen receptor alfa; E1, oestrone; E2, oestradiol; EGFR, epidermal growth factor receptor; ERβ, estrogen receptor beta; FAK, focal adhesion kinase; HERB2, human epidermal growth factor receptor 2; HERB3, human epidermal growth factor receptor 3; IGFR, insulin-like growth factor receptor; LIMK2, LIM domain kinase 2; LPA, lysophosphatidic acid; mAR, membrane androgen receptors; MATs, microtubule-targeting agents; MT, microtubule; MTSAs, microtubule-stabilizing agents; mTOR, mammalian target of rapamycin; PKC, protein kinase C; PLC, phospholipase C; PLD, phospholipase; ProB2, procyanidin B2; ProB, procyanidins; PI3K, phosphoinositide-3-kinase; RTK, receptor tyrosine kinase; ROCK, rho-associated protein kinase; T, testosterone.

Such a difference could be related to the molecular characteristics of cells as well as the compounds characteristics, particularly variation in the cell ability to deal with estrogen, progesterone and androgen activities modulated by the compounds tested (Figure 12). It is postulated that some types of CATs have binding affinity for ERβ, ERα, PR and/or AR and could exhibit similar effects to that of selective modulators of hormone receptors, such as ER modulators (SERMs) [66,67,68,69,70], selective estrogen down-regulators (SERDs) [71,72,73], selective progesterone receptors modulators (SPRMs) [72] and selective androgen receptor modulators (SARMs), even in androgen-independent PC [74,75]. Furthermore, it is suggested that CATs can inhibit different enzymes involved in estrogen and androgen synthesis such as aromatase (aromatase inhibitors (AIs)] [76,77,78,79]) and 5α-reductase [80,81]. Likewise, it seems that CATs exhibit a pleiotropic effect on several molecular targets (Figure 12A–C):

- It is considered that the binding affinity of CATs to ERβ and ERα is likely conditioned by the structure and the dose as well as the cell type [66,68,69,72]. CAT it is a flavan-3-ol, whose family is considered structurally similar to isoflavones that exhibit some structural similarities with 17-b-estradiol as well as with other steroid hormones and steroid hormone antagonists. Thus, CAT could act as an ER agonist or antagonist whose action is both ER concentration- and ER isomorph-dependent or flavane type-dependent. The number of hydroxyl groups, mainly those in the flavonoid B-ring, appeared to be of importance, while changes in the A- or B-hydroxylation rings are given minor importance [82,83].
- Additionally, it is suggested that some CATs act as ER down-regulators [71,72,73], particularly through ERα36 in TNBC progenitor cells [84]. In addition, it is suggested that some CATs can indirectly down-regulate ERα [73] via PR [72] in MCF-7 cells.
- Different CATs can antagonize androgen, resulting in a decrease in AR-mediated transcriptional activation [74]. Furthermore, Kampa et al. [75] suggested that flavanol dimers B1-B4 (oligomeric Pro) and in particular, oleylated B2 could be considered a therapeutic agent for advanced PC since it had a powerful agonist effect on membrane AR in androgen-independent DU145 PC cells (ProB2 > ProB3 = ProB4 > ProB1).
- In the present study, we observed that MCF-7 and DU145 cells were most sensitive to ProB2 and/or CAT than the remaining BC and PC cells, respectively. It is known that MCF-7 cells and T47D cells both are ER+, PR+ AR+ cells, but MCF-7 cells differ from T47D cells in that they continually express ER while T47D cells lose ER and PR expression under estrogen withdrawal [85] as well as in ER and PR expression levels [29,86]. Additionally, AR activity is necessary for ER+/AR+ MCF-7 and T47D cell line growth [87]. MCF-7 and T47D both express AR, but two different regulatory pathways may be involved in the androgen-induced stimulation of proliferation, resulting in different repercussions on proliferative activity. MCF-7 cells show AR-mediated and AR-independent MCF-7 mechanisms, whereas T47D exhibits an AR-mediated one. In addition, MCF-7 cells express a wild-type AR characterized by a shortened CAG repeat, while T47D presents a CAG repeat length considered in the normal range [88]. This shortening represents a more active receptor [89]. In addition, these cell lines differ in AR-FL (full-length AR) and splice variants from the AR gene (ARVs) expression. The latter are constitutively active androgen-independent transcription factors that are implicated in resistance to treatment in PC and in BC. ARVs vary widely among the BC and PC cell lines tested. As an example, MDA-MB-231 cells exhibits the highest ARV to AR-FL ratio, followed by T47D and MCF-7 cells. Additionally, MDA-MB-231 cells exhibit low AR-FL and high AR-V3 expression. AR45, AR-V1, AR-V7 and AR-V9 splice variants rather than T47D and MCF-7 cells [90]. On the other hand, DU145 and PC3 cells do not express AR and Erβ, while PC3 express ERα [91].
- Additionally, it should be remembered that MCF7 and T47D are PR+. Regarding PC cells lines used in the present study, PC3 and DU145 differ in the expression of PR isoforms (isoforms A and B). While PC3 presents the two promotors methylated and inactivated, DU145 presents them unmethylated and activated. It should be noted that two promoters control the expression of PR isoforms and hypermethylation of cytosine-rich areas in promoters, which is considered a functional inactivation [92]. The overexpression of the isoform A is involved in cyclin D1 activity; proliferative promoters increase (TGFβ1) as well as pro-invasive and pro-migratory changes (basal membrane disruption, etc.). Interestingly, those effects can be counteracted by antiestrogens [93].
- Grape seed extracts and red wine, and specifically ProB2 dimers (from grape seeds as well as red wine), could suppress aromatase activity [76,77,78,79], inducing hormonal changes attributed to AI [79]. DU145 and PC3 cells used in the present study have constitutive aromatase activity, but with different levels [94]. Regarding BC cell lines, there are contradictory data on aromatase expression, with the presence [95,96] or absence of this enzyme [97]. In the present study, we found that PC and BC cell lines show different degrees of susceptibility to the effects of CAT and ProB2.
- 5α-reductase 1 is present in both PC cell lines used in this study, while 5α-reductase 2 is present in DU145 PC cells [98]. With respect to BC cell lines, MCF-7 and T47D BC cells used in this study both express 5α-reductase 1 [99]. It was suggested that some CATs from green tea seed extract exert 5α-reductase inhibitory characteristics. On the other hand, it seems that natural compounds that act as inhibitors possessing a catechol group have selectivity for the type 1 isozyme [80]. Additionally, polymeric anthocyanins seem to be type 2 isozyme inhibitors [81]. Thereby, it was suggested that they are useful for the prevention as well as the treatment of androgen-dependent disorders [80].

According to the above mentioned, the effects of CAT and ProB2 would be expected to be greater in MCF-7 cells and DU145 cells compared to the remaining BC and PC cell lines used, as we observed. An important issue for our future research is the effect of blocking of the AR, ER and PR on the ability of the tested compounds to influence their antitumor efficacy on BC and PC cells.

The second question in this research was to establish the efficacy of co-adjuvant therapy with CAT or ProB2 plus DOCE. In regard to this question, this study found that ProB2 sensitizes only one PC cell line (DU145) to DOCE effects. Herein, we report that the combination therapy with ProB2 and DOCE exhibited a much stronger anti-proliferative and anti-apoptotic effect (from three-fold to six-fold increase) on DU145 cells as compared to individual DOCE treatment. On the other hand, we observed that ProB2 and CAT sensitize all the BC cells tested (cell-independent) to the anti-proliferative effect of DOCE. Nevertheless, sensitization to DOCE apoptotic effects was cell-dependent. Hence, both ProB2 and CAT sensitize MCF-7 cells to the pro-apoptotic effect of DOCE. By contrast, only one of these compounds caused this effect on T47D (ProB2) and MDA-MB-231 (CAT) cells.

**Figure 13:**
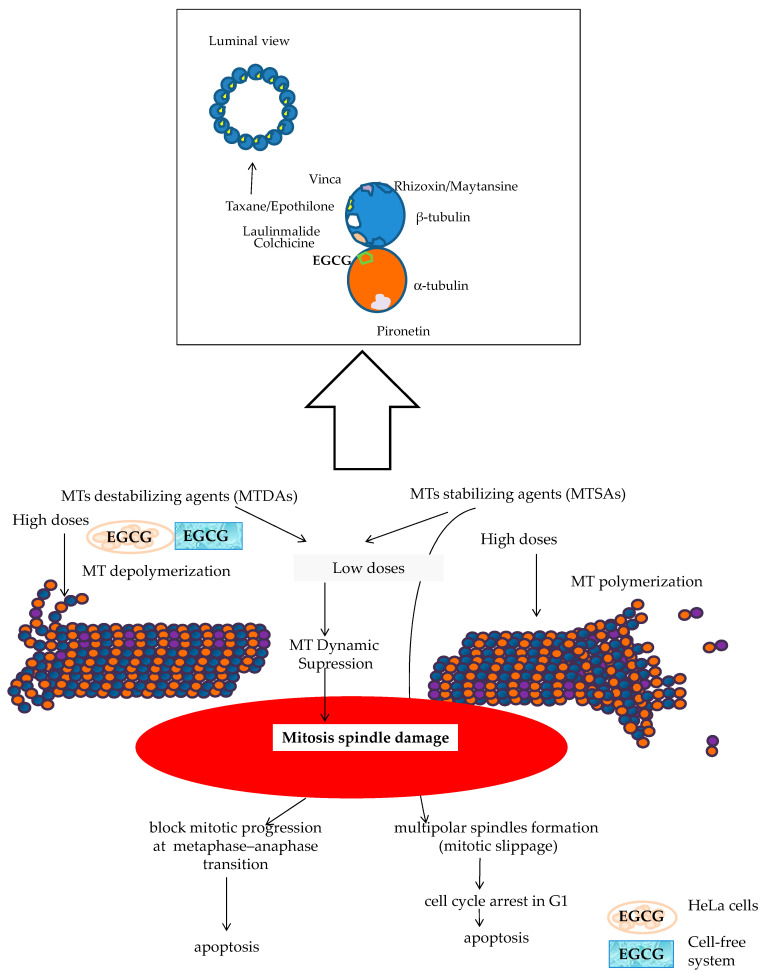
Types of MTAs according to their effect on microtubules: MTDAs and MTSAs. MTDAs and MTSAs induce depolarization and polarization of microtubules, respectively, at high doses, whereas both MTAs cause suppression of MTs dynamics at low doses [105]. Main MTDAs- and MTSAs-domain binding drugs are showed. The schematic representation of drug-binding sites on tubulin is based on McLoughlin et al. [119] with modifications. CATs could interact with tubulin [107,108,109]. Based on in silico data, it appeared that some CATs bind to the α-subunit of tubulin, close to the colchicine site [108]. DOCE binds to the β-tubulin subunits of microtubules [124], at taxane-binding side, inducing suppression of MTs dynamics (low doses) or stabilization of microtubules, and thereafter cell-cycle arrest effects [105]. Abbreviations: EGCG, epigallocatechin gallate; MT, microtubule; MTDAs, microtubule-destabilizing agents; MTSAs, microtubule-stabilizing agents.

An interesting question is whether the sensitization by ProB2 and CAT to DOCE on MCF-7 cells occurs. To demarcate some possibilities:

- Combined therapy with receptor modulators and chemotherapy drugs such as DOCE has long since been given improving clinical outcomes (CHAARTED trial, STAMPEDE trial, PROXIMA trial, etc.) [59,100,101,102,103]. Moreover, it has recently been found that SERMs can act as modulators of microtubules at taxane sites [104]. As it is shown in Figure 12A–C, ProB2 and/or CAT could exert a pleiotropic effect on hormone receptors and hormone synthesis, in particular in ER+PR+, AR+ BC cells such as MCF-7 cells (already discussed above). Additionally, as it is shown in Figure 12A, CATs and DOCE have some similar mechanisms of action such as AIs or AR modulators. These aspects may have contributed to the sensitizing effect of DOCE in the combined treatment of ProB2 plus DOCE and CAT plus DOCE shown in this study.
- The dual targeting of the microtubule structure could sensitize cancer cells to chemotherapy treatment (Figure 13). Microtubule-targeting agents (MTAs) such as DOCE are an effective treatment for solid tumors such as BC and PC. This drug is the standard treatment for BC and PC, both metastatic and resistant to other treatments [59,60,101,102,103]. A large number of naturally sourced MTAs are antimitotic agents by acting on tubulin protein and its polymers microtubules [105,106]. These drugs can be classified according to their effect in microtubule-destabilizing agents ((MTDAs) vinca alcaloids, cryptophycins, etc.) and microtubule-stabilizing agents (MTSAs) that include Paclitaxel and its semisynthetic analogue DOCE [105], the chemotherapy drug tested in the present study. DOCE binds to the β-tubulin subunits of microtubules at the taxane-binding side, inducing the suppression of MT dynamics (low doses) or the stabilization of microtubules, and thereafter, cell-cycle arrest [105].
- The molecules tested in our study could act as MTAs, and their administration with DOCE could contribute to the sensitization to DOCE by CAT and ProB2 observed in our study (Figure 13). Recent reports suggest that natural compounds such as CATs can exert anti-proliferative activity against parasites [107] and lung, cervix [108] and hepatoma cancer [109] cell lines could be mediated by union to β-tubulin chain [103], α-tubulin protein and α-β heterodimer [109]. In silico modeling has provided additional insights into potential mechanisms of some types of CATs by binding to α-tubulin molecules at the interface, between α-and β-heterodimers, which could be responsible for the depolymerization of MTs in cervix cancer cells. Additionally, a cell-free system study showed that EGCG causes the inhibition of microtubule polymerization [108]. In our future research, we will analyze the effect of ProB2 and CAT, with and without DOCE, on α-and β-tubulin as well as microtubules.
- Among the cells used in this study, the MCF-7 cell line has been shown to be the most sensitive to treatment. Overall, our results suggest that CDKN1A, BAX, CASP9 and E-cadherin up-regulated expression contributing to the ProB2- and CAT-induced sensitization to DOCE in MCF-7 cells, since:
  1. CDK 1 is of particular importance as it is essential to ensure cell cycle progression and apoptosis [110,111,112]. CDK inhibitors had been postulated as a promising therapeutic strategy for advanced cancer [113]. CDKN1A suppresses tumor growth and apoptosis [41,42,111,113]. It is postulated that preventing the up-regulation of the CDK1 axis improves the sensitivity of cancer cells to chemotherapeutic agents, increasing their effectiveness. Combined treatment with CDK inhibitors and chemotherapeutic agents significantly increases the effectiveness of the chemotherapeutic agent [108,109,110,111,112,113,114,115], including docetaxel [114]. In addition, inactivating CDK1 agents can induce long-term cytotoxicity (clonogenic assay) [116]. In the present study, we observed that MCF-7 cells treated with CAT plus DOCE and ProB2 plus DOCE caused a high increase in CDK1 inhibitor. Additionally, in this study, the combined treatment of PRoB2 plus DOCE as well as CAT plus DOCE inhibited the formation of colonies drastically in relation to DOCE alone.
  2. BAX and CASP9 promote apoptosis [43,44,45,46,47] and both CASP3 and CASP9 seem to be involved in taxane-induced cell death [46]. Interestingly, we found that both BAX (≈ eight-fold) and (≈ two-fold) increased in CAT plus DOCE-treated cells and ProB2 plus DOCE-treated cells, respectively, in relation to DOCE.
  3. E-cadherine, an epithelial phenotype maintaining gene, can inhibit mitogenic signaling [48] and its suppression is associated to DOCE chemoresistance in hormonal tumors [49,50,53]. As far as we observed, with a great increase in E-cadherine under combined treatment compared to DOCE single treatment, a highly sensitizing effect could be expected.
  4. Sensitization to DOCE induced by the compounds tested on MCF-7 cells does not appear to be influenced by the expression of beta tubulin III (TUBB3), baculoviral inhibitor of apoptosis repeat-containing 5 (BIRC5) and forkhead box protein P3 (FOXOP3). It is known that FOXOP3 is involved in apoptosis [117] and that BIRC5 is a bi-functional protein that acts not only as a mitotic regulator but also inhibits caspase activation [118].

Taken together, these results suggest that these anti-tumor therapeutic effects with minimal toxic effects in healthy cells could be useful in clinical practice. However, caution must be applied, as more studies are required in this regard.

## 4. Material and Methods

### 4.1. Cell Culture

Human BC cell lines MCF-7 (cat. no. HTB-22), T47D (cat. no. HTB-133), MDA-MB-231 (cat. no. HTB-26), as well as PC cell lines PC3 (cat. no. CRL-1435) and DU145 (cat. no. HTB-81) (Table 3), and also healthy primary mammary epithelial cells (HMEC, PCS-600-010) and epithelial prostate cells (HprEC, PCS-440-010) were obtained from ATCC (ATCC; Manassas, VA, USA) and cultured according to their recommendations (culture conditions, culture media and antimicrobials/antimycotics). Stock solutions of CAT (cat. no. 43412, Sigma-Aldrich, Madrid, CM, Spain), ProB2 (cat. no. 42157, Sigma-Aldrich, Madrid, CM, Spain) and DOCE (cat. no. 01885, Sigma-Aldrich, Madrid, CM, Spain) were prepared in dimethyl sulfoxide (DMSO, cat. no. D2650; Sigma-Aldrich, Madrid, CM, Spain) and diluted with complete medium. The controls had an equal volume of DMSO (final concentration < 0.05%).

### 4.2. Treatments

DOCE doses were chosen according to their anti-tumor effects on previous reports [126]. The selected doses of CAT and ProB2 were chosen according to the toxicity levels of CAT and ProB2 administration on healthy prostate cells (Table 1A) and healthy mammary cells (Table 1B). Survival was dramatically decreased with doses up to 50 µM (differences *p* < 0.05).

The following treatments were used: fresh complete medium containing DMSO at final concentration < 0.05% (controls); CAT at 5, 20, 30 and 50 µM (MCF-7, T47D and MDA-MB-231 cells); ProB2 at 10, 25, 50 µM (MDA-MB-231 and MCF-7 cells); ProB2 at 0.1, 0.5, 1, 10 µM (T47D cells) and/or DOCE at 60, 120 nM (MCF-7 cells) and/or DOCE at 50, 100 nM (MDA-MB-231 and T47 cells); CAT at 50, 70, 80 y 100 μM (PC3 and DU145 cells); ProB2 at 25, 50, 100, 150 μM (PC3 and DU145 cells) and DOCE at 1, 2 nM (PC3 and DU145 cells).

### 4.3. Viability Assay

Cells were seeded in 96-well plates (5 × 10^3^) overnight before exposure to the treatments. After incubation under standard culture conditions (37 °C, humidified atmosphere of 95% air, 5% CO2) for 24, 48 or 72 h, the numbers of viable cells were evaluated using trypan blue (cat. no. T6146, Sigma-Aldrich, Madrid, CM, Spain) dye exclusion [127].

### 4.4. Sensitization of Cancer Cells to Growth Suppression by DOCE

The combination effect between DOCE and CAT and DOCE and ProB2 was calculated as previously described [38].

### 4.5. Apoptosis Assay

The cells were seeded (2 × 10^4^) on coverslips overnight before exposure to the treatments (DMSO, CAT, ProB2 and/or DOCE at the same concentrations mentioned above). After incubation (at 37 °C for 72 h), the cells were stained as previously described [126]. The apoptotic cells were counted under fluorescence microscope (40×) [125].

### 4.6. Colony Formation Assay

In order to evaluate the cytotoxic effects of treatment over a prolonged period of time, clonogenic assays were performed on the cell lines 14 days after treatment. The cells were seeded (1000 cells/well by quadrupling) in 6-well plates before exposure to the treatments [39]. The cells were observed during the experiment and at day 14, colonies with more than 50 cells were counted under a phase contrast microscope. Finally, the colonies were dyed with violet crystal (cat. no. C6158; Sigma-Aldrich, Madrid, MD, Spain) and counted through the Digital Analysis Program Cell Profile.

### 4.7. Cell Migration Assay

DU145 and MCF-7 cells (1 × 10^5^) treated with ProB2 or CAT were suspended in 100 µL of serum-free medium and seeded into the upper chamber of Millicell Cell Culture Inserts for 24-well plates (cat. no. PIEP12R48; Merck Millipore Madrid, MD, Spain). Later, their migration ability was determined as previously described [126,128].

### 4.8. Reverse Transcription-Quantitative Polymerase Chain Reaction (RT-qPCR)

Considering that MCF cells were the most sensitive cancer cells to both CAT and ProB2 effects, to determine the molecular mechanism by which these compounds decrease proliferative activity, clonogenic activity and migration as well as sensitize BC to DOCE, we preformed RT-qPCR.

After the cells (1 × 10^4^ cells/well) were grown in growth chambers (cat. no. C6932; Sigma-Aldrich, Madrid, MD, Spain) for 24 h, they were treated with CAT, ProB2, and/or DOCE and cultured during 72 h. RNAs were isolated from cells using an RNAspin Mini RNA Isolation Kit (cat. no. MRN70, Sigma-Aldrich, Madrid, MD, Spain). Real-time PCR reactions were performed as previously described [126]. Three independent analyses were performed for each sample, and changes in the expression of target expression of genes normalized to GAPDH were calculated with the ∆∆Cq method for relative quantification and expressed as the fold change relative to the untreated control. RT-PCR primer sets were purchased from Sigma-Aldrich.

### 4.9. Statistical Analysis

The results represent the mean of at least three independent experiments (mean ± SD). The significance of difference in measured variables between the control and treated groups was studied by the t-test or ANOVA. Difference was considered significant at *p* ≤ 0.05.

## Figures and Tables

**Table 1:** Effect of ProB2 and CAT on proliferation of (A) healthy prostate and (B) healthy mammary cells (72 h).

(A)							
**DOCE (nM)**	**ProB2 (µM)**	**Mean**	**SD**	**DOCE (nM)**	**CAT(µM)**	**Mean**	**SD**
0	0	98.84	0.87	0	0	98.01	0.87
0	25	98.51	0.92	0	50	99.6	0.79
0	50	97.83	0.65	0	70	98.87	0.88
0	100	97.75	1.03	0	80	98.32	0.86
0	150	96.62	0.78	0	100	99.05	0.68
0	200	32.22 *	0.93	0	150	32.8 *	1.15
1	0	99.11	1.11	1	0	97.87	0.5
1	25	99.27	1.06	1	50	98.67	0.74
1	50	98.08	0.93	1	70	98.86	0.87
1	100	98.45	0.68	1	80	99.34	0.75
1	150	97.9	0.86	1	100	98.43	0.67
1	200	27.86 *	0.92	1	150	24.32 *	0.87
2	0	98.33	1.21	2	0	97.08	1.21
2	25	98.08	1.23	2	50	98.06	1.13
2	50	99.43	0.96	2	70	97.97	0.94
2	100	98.52	0.87	2	80	98.87	0.89
2	150	98.31	0.79	2	100	99.56	1.27
2	200	24.12 *	0.93	2	150	21.13 *	0.83
(B)							
**DOCE (nM)**	**ProB2 (µM)**	**Mean**	**SD**	**DOCE (nM)**	**CAT (µM)**	**Mean**	**SD**
0	0	97.32	0.61	0	0	98.33	1.17
0	10	98.91	0.87	0	5	97.09	1.72
0	25	98.10	1.13	0	20	99.93	1.22
0	50	97.02	0.65	0	30	97.53	0.96
0	60	42.21 *	0.83	0	50	98.04	0.78
0	80	31.01 *	1.29	60	0	97.13	1.04
0	100	25.47 *	1.09	60	5	97.21	1.62
60	0	95.31	1.24	60	20	97.22	1.77
60	10	96.04	1.20	60	30	98.28	0.94
60	25	96.92	1.28	60	50	97.67	0.79
60	50	97.31	0.76	120	0	98.75	1.65
60	60	37.06 *	0.56	120	5	98.59	1.31
60	80	29.23 *	0.72	120	20	98.81	0.84
60	100	14.26 *	0.37	120	30	98.01	1.52
120	0	96.72	1.36	120	50	97.27	1.19
120	10	97.44	1.03				
120	25	97.03	0.74				
120	50	96.15	0.82				
120	60	35.24 *	0.59				
120	80	22.97 *	0.43				
120	100	13.28 *	0.47				

Proliferative activity detected using a trypan blue dye exclusion assay. The data are means ± SD (*n* = 3). Significantly different compared with control, * *p* < 0.05. Abbreviations: CAT, catechin; DOCE, docetaxel; ProB2, ProB2, procyanidin B2.

**Table 2:** Gene list that represents at least 1.5-fold of change of genes encoding cell proliferation-, apoptosis- and EMT-related proteins in MCF-7 cells treated either with ProB2 (50 µM), CAT (50 µM), DOCE (120 nM), ProB2 (50 µM)+ DOCE (120 nM) or CAT (50 µM)+ DOCE (120 nM) for 72 h.

Gene Symbol *	Description ^†^	Treatment	Related to Control	Related to DOCE
CDH1	Cadherin 1, type 1, E-cadherin (epithelial)	DOCE	0.00770	
		CAT	0.51759	67.19063 ^a^
		ProB2	0.10638	13.68735 ^a^
		CAT+DOCE	0.30540	39.51263 ^a^
		ProB2+DOCE	0.07310	9.49801 ^a^
CDKN1A	Cyclin-dependent kinase inhibitor 1A	DOCE	5.06 ^a^	
		CAT	1.60 ^a^	0.97
		ProB2	0.22	7.39 ^b^
		CAT+DOCE	0.38	8.22 ^b^
		ProB2+DOCE	0.49	2.40 ^b^
BAX	BCL-2-associated X protein	DOCE	0.05	
		CAT	0.01	0.02
		ProB2	0.15	0.37
		CAT+DOCE	7.25 ^a^	8.46 ^b^
		ProB2DOCE	1.37	1.92 ^b^
CASP9	Caspase-9	DOCE	18.02465 ^a^	
		CAT	1.55971 ^a^	0.08442
		ProB2	0.36939	0.02025
		CAT+DOCE	8.27195 ^a^	0.46528
		ProB2+DOCE	1.48718 ^a^	0.08252

A value greater than 1.5 indicates an increase in expression. Data are the mean of 3 independent experiments ± SE. ^a^, *p* < 0.05, significantly different compared with control treatment; ^b^, *p* < 0.05, significantly different compared with DOCE (120nM) treatment. Abbreviations: BAX, BCL-2-associated X protein; CDKN1A, cyclin-dependent kinase inhibitor 1A; CASP9, caspase-9; CAT, catechin; CDH1, cadherin type 1, E-cadherin; EMT, epithelial-mesenchymal transition. * Genecards, human gene database [54]; ^†^ Gene-NCBI [55].

**Table 3:** Characteristics of cancer cell lines.

Cell Line	T. Type ^a^	T. source	Pan-Can	IHC-ST ^b^	Tx-st	Diffs	TP53 Status ^c^	ER/PR/HERB2/AR ^d^
MCF-7	A, NOS	PE	Lum/HER2	A	Lum	Lum1	Wt	ER +/PR+/HERB2-/AR+
T47D	Idc, NOS	PE	Lum/HER2	A	Lum	Lum2	m	ER+/PR+/HERB2-/AR+
MDA-MB-231	A, NOS	PE	------	C	Claudin low	------	m	ER-/PR-/HERB2-/AR+
PC3	AD, Grade IV	Bom-d	------	------	------	------	m	ER/PR/AR-
DU145	AD, NOS	Brm-d	------	------	------	------	m	ER/PRAR-

[28,29,30,31,32,33,34,35,36,37,54,55,86,88,89,90,125]. Abbreviations: A, adenocarcinoma; ARs, androgen receptor status; Bom-d, bone metastasis-derived; Brm-d, brain metastasis-derived; Diffs, differentiation status; Idc, infiltrating duct carcinoma; IHC-ST, immunohistochemistry subtype; m, mutant; NOS, not otherwise specified; Pan-Can ST, nanoString Pan Cancer Pathways panel; PE, pleural effusion; T type, tumor type; T. source, tissue source; Tx-ST, transcriptome subtype; w, wild type. ^a^ Tumor type, including morphology and/or histology based on International Diseases on Oncology (ICD-O), World Health Organization. International classification of diseases for oncology (ICD-O)–3rd edition, 1st revision and SNOMED. C50.- ICD-O topography. C61.- ICD-O topography [29,30]. ^b^ IHC-ST:A,E-Cad+/Vim-/ER+/HER2+/AR+/EGFR+/;B,E-Cad+/Vim-/ER-/HER2+/AR+/EGFR+;C,E-Cad-/Vim+/ER-/HER2-/AR-/CK19+/EGFR+ [36]. ^c^ International Agency for Research on Cancer (IARC); Catalog of Somatic Mutations in Cancer (COSMIC) [30,36,125]. ^d^ Expression level: [29], AR high [123]; T47D, PR high [29]; ER, MCF7 > T47D; PR, T47D > MCF7 [86]; AR, MCF7 > T47D > MDA-MB-231 [86]; PC3, ERα-/ERβ-; DU145, ERα-/ERβ+ [91]; PC3 PR-A and PR-B promotors methylated and inactivated DU145 presents them unmethylated and activated [89].

## Data Availability

The authors confirm that the data supporting the findings of this study are available within the article.

## References

[1] Risbridger G.P., Davis I.D., Birrell S.N., Tilley W. (2010). Breast and prostate cancer: More similar than different. Nat. Rev. Cancer.

[2] Misawa A., Inoue S. (2015). Estrogen-Related Receptors in Breast Cancer and Prostate Cancer. Front. Endocrinol..

[3] Bray F., Ferlay J., Soerjomataram I., Siegel R.L., Torre L.A., Jemal A. (2018). Global cancer statistics 2018: GLOBOCAN estimates of incidence and mortality worldwide for 36 cancers in 185 countries. CA Cancer J. Clin..

[4] Lamy P.-J., Trétarre B., Rébillard X., Sanchez M., Cénée S., Ménégaux F. (2018). Family history of breast cancer increases the risk of prostate cancer: Results from the EPICAP study. Oncotarget.

[5] Frank C., Sundquist J., Hemminki A., Hemminki K. (2017). Familial Associations between Prostate Cancer and Other Cancers. Eur. Urol..

[6] Harbeck N., Penault-Llorca F., Cortes J., Gnant M., Houssami N., Poortmans P., Ruddy K., Tsang J., Cardoso F. (2019). Breast cancer. Nat. Rev. Dis. Primers..

[7] Zardavas D., Irrthum A., Swanton C., Piccart M. (2015). Clinical management of breast cancer heterogeneity. Nat. Rev. Clin. Oncol..

[8] Bertucci F., Finetti P., Goncalves A., Birnbaum D. (2020). The therapeutic response of ER+/HER2− breast cancers differs accord-ing to the molecular Basal or Luminal subtype. NPJ Breast Cancer.

[9] Toss A., Cristofanilli M. (2015). Molecular characterization and targeted therapeutic approaches in breast cancer. Breast Cancer Res..

[10] Venema C.M., Bense R.D., Steenbruggen T.G., Nienhuis H.H., Qiu S.Q., van Kruchten M., Brown M., Tamimi R.M., Hospers G.A., Schröder C.P. (2019). Consideration of breast cancer subtype in targeting the androgen receptor. Pharmacol. Ther..

[11] Karantanos T., Corn P.G., Thompson T.C. (2013). Prostate cancer progression after androgen deprivation therapy: Mechanisms of castrate resistance and novel therapeutic approaches. Oncogene.

[12] Tolkach Y., Kristiansen G. (2018). The Heterogeneity of Prostate Cancer: A Practical Approach. Pathobiology.

[13] Grindstad T., Richardsen E., Andersen S., Skjefstad K., Khanehkenari M.R., Donnem T., Ness N., Nordby Y., Bremnes R.M., Al-Saad S. (2018). Progesterone Receptors in Prostate Cancer: Progesterone receptor B is the isoform associated with disease progression. Sci. Rep..

[14] Di Zazzo E., Galasso G., Giovannelli P., Di Donato M., Castoria G. (2018). Estrogens and Their Receptors in Prostate Cancer: Therapeutic Implications. Front. Oncol..

[15] Hwang C. (2012). Overcoming docetaxel resistance in prostate cancer: A perspective review. Ther. Adv. Med Oncol..

[16] Murray S., Briasoulis E., Linardou H., Bafaloukos D., Papadimitriou C. (2012). Taxane resistance in breast cancer: Mechanisms, predictive biomarkers and circumvention strategies. Cancer Treat. Rev..

[17] Nurgali K., Jagoe R.T., Abalo R. (2018). Editorial: Adverse Effects of Cancer Chemotherapy: Anything New to Improve Tolerance and Reduce Sequelae?. Front. Pharmacol..

[18] Lin S.R., Chang C.H., Hsu C.F., Tsai M.J., Cheng H., Leong M.K., Sung P.J., Chen J.C., Weng C.F. (2020). Natural compounds as potential adju-vants to cancer therapy: Preclinical evidence. Br. J. Pharmacol..

[19] de la Iglesia R., Milagro F.I., Campión J., Boqué N., Martíne J.A. (2010). Healthy properties of proanthocyanidins. Biofactors.

[20] Rauf A., Imran M., Abu-Izneid T., Haq I.-U., Patel S., Pan X., Naz S., Silva A.S., Saeed F., Suleria H.A.R. (2019). Proanthocyanidins: A comprehensive review. Biomed. Pharmacother..

[21] Kumar N.B., Pow-Sang J., Egan K.M., Spiess P.E., Dickinson S., Salup R., Helal M., McLarty J., Williams C.R., Schreiber F. (2015). Randomized, Placebo-Controlled Trial of Green Tea Catechins for Prostate Cancer Prevention. Cancer Prev. Res..

[22] Cui K., Li X., Du Y., Tang X., Arai S., Geng Y., Xi Y., Xu H., Zhou Y., Ma W. (2017). Chemoprevention of prostate cancer in men with high-grade prostatic intraepithelial neoplasia (HGPIN): A systematic review and adjusted indirect treatment comparison. Oncotarget.

[23] Sawada N. (2017). Risk and preventive factors for prostate cancer in Japan: The Japan Public Health Center-based prospective (JPHC) study. J. Epidemiol..

[24] Gu L., Kelm M.A., Hammerstone J.F., Beecher G., Holden J., Haytowitz D., Gebhardt S., Prior R.L. (2004). Concentrations of Proanthocyanidins in Common Foods and Estimations of Normal Consumption. J. Nutr..

[25] Gu L., Kelm M.A., Hammerstone J.F., Beecher G., Holden J., Haytowitz D., Prior R.L. (2003). Screening of foods containing proantho-cyanidins and their structural characterization using LC-MS/MS and thiolytic degradation. J. Agric. Food Chem..

[26] Araujo F., Nitzke J.A., Blauth C., Vogt E. (2010). Chocolate and red wine–A comparison between flavonoids content. Food Chem..

[27] He Z., Zhang J., Yuan X., Xi J., Liu Z., Zhang Y. (2019). Stratification of Breast Cancer by Integrating Gene Expression Data and Clinical Variables. Molecules.

[28] Goodspeed A., Heiser L.M., Gray J., Costello J.C. (2016). Tumor-Derived Cell Lines as Molecular Models of Cancer Phar-macogenomics. Mol. Cancer Res..

[29] The Broad Institute Cancer Cell Line Encyclopedia (CCLE) (2021). The Broad Institute of MIT&Harvad. ttp://www.broadinstitute.org/ccle/home.

[30] Welcome—IARC TP53 Database. https://p53.iarc.fr/.

[31] Moran D.M., Mak C.G. (2010). Nutlin-3a induces cytoskeletal rearrangement and inhibits the migration and invasion capacity of p53 wild-type cancer cells. Mol. Cancer Ther..

[32] Concin N., Zeillinger C., Tong D., Stimpfl M., König M., Print D., Concin N., Zeillinger C., Tong D., Stimpfl M. (2003). Comparison of p53 mutational status with mRNA and protein expression in a panel of 24 human breast carcinoma cell lines. Breast Cancer Res. Treat..

[33] Dias K., Dvorkin-Gheva A., Hallett R.M., Wu Y., Hassell J., Pond G.R., Levine M., Whelan T., Bane A.L. (2017). Claudin-Low Breast Cancer; Clinical & Patho-logical Characteristics. PLoS ONE.

[34] Jiang G., Zhang S., Yazdanparast A., Li M., Pawar A.V., Liu Y., Inavolu S.M., Cheng L. (2016). Comprehensive comparison of molecular portraits between cell lines and tumors in breast cancer. BMC Genom..

[35] Barretina J., Caponigro G., Stransky N., Venkatesan K., Margolin A.A., Kim S., Wilson C.J., Lehár J., Kryukov G.V., Sonkin D. (2012). The Cancer Cell Line Encyclopedia enables predictive modelling of anticancer drug sensitivity. Nature.

[36] Saunus J.M., Smart C.E., Kutasovic J.R., Johnston R., Croft P.K.-D., Miranda M., Rozali E.N., Vargas A.C., Reid L.E., Lorsy E. (2017). Multidimensional phenotyping of breast cancer cell lines to guide preclinical research. Breast Cancer Res. Treat..

[37] Neve R.M., Chin K., Fridlyand J., Yeh J., Baehner F.L., Fevr T., Clark L., Bayani N., Coppe J.-P., Tong F. (2006). A collection of breast cancer cell lines for the study of functionally distinct cancer subtypes. Cancer Cell.

[38] Lee H.Y., Oh S.H., Suh Y.A., Baek J.H., Papadimitrakopoulou V., Huang S., Hong W.K. (2005). Response of non-small cell lung cancer cells to the inhibitors of phosphatidylinositol 3-kinase/Akt- and MAPK kinase 4/c-Jun NH2-terminal kinase pathways: An ef-fective therapeutic strategy for lung cancer. Clin. Cancer Res..

[39] Franken N.A.P., Rodermond H.M., Stap J., Haveman J., Van Bree C. (2006). Clonogenic assay of cells in vitro. Nat. Protoc..

[40] Gandalovičová A., Rosel D., Fernandes M., Veselý P., Heneberg P., Čermák V., Petruzelka L., Kumar S., Sanz-Moreno V., Brábek J. (2017). Migrastatics—Anti-metastatic and Anti-invasion Drugs: Promises and Challenges. Trends Cancer.

[41] Abbas T., Dutta A. (2009). p21 in cancer: Intricate networks and multiple activities. Nat. Rev. Cancer.

[42] Liao X.-H., Lu D.-L., Wang N., Liu L.-Y., Wang Y., Li Y.-Q., Yan T.-B., Sun X.-G., Hu P., Zhang T.-C. (2014). Estrogen receptor α mediates proliferation of breast cancer MCF-7 cells via a p21/PCNA/E2F1-dependent pathway. FEBS J..

[43] Westphal D., Kluck R., Dewson G. (2014). Building blocks of the apoptotic pore: How Bax andBak are activated and oligomerize during apoptosis. Cell Death Differ..

[44] Kalkavan H., Green D. (2018). MOMP, cell suicide as a BCL-2 family business. Cell Death Differ..

[45] Delbridge A.R.D., Strasser A. (2015). The BCL-2 protein family, BH3-mimetics and cancer therapy. Cell Death Differ..

[46] Jelínek M., Balušíková K., Schmiedlová M., Němcová-Fürstová V., Šrámek J., Stančíková J., Zanardi I., Ojima I., Kovář J. (2015). The role of individual caspases in cell death induction by taxanes in breast cancer cells. Cancer Cell Int..

[47] Wang S., He M., Li L., Liang Z., Zou Z., Tao A. (2016). Cell-in-Cell Death Is Not Restricted by Caspase-3 Deficiency in MCF-7 Cells. J. Breast Cancer.

[48] Jeanes A., Gottardi C., Yap A. (2008). Cadherins and cancer: How does cadherin dysfunction promote tumor progression?. On-Cogene.

[49] Marín-Aguilera M., Codony-Servat J., Reig Ò., Lozano J.J., Fernández P.L., Pereira M.V., Jiménez N., Donovan M., Puig P., Mengual L. (2014). Epithelial-to-Mesenchymal Transition Mediates Docetaxel Resistance and High Risk of Relapse in Prostate Cancer. Mol. Cancer Ther..

[50] Işeri O.D., Kars M.D., Arpaci F., Atalay C., Pak I., Gündüz U. (2011). Drug resistant MCF-7 cells exhibit epithelial-mesenchymal transition gene expression pattern. Biomed. Pharmacother..

[51] Fares J., Fares M.Y., Khachfe H.H., Salhab H.A., Fares Y. (2020). Molecular principles of metastasis: A hallmark of cancer revisit-ed. Sig. Transduct. Target Ther..

[52] Olea-Flores M., Juárez-Cruz J.C., Mendoza-Catalán M.A., Padilla-Benavides T., Navarro-Tito N. (2018). Signaling Pathways In-duced by Leptin during Epithelial⁻Mesenchymal Transition in Breast Cancer. Int. J. Mol. Sci..

[53] Li W.-J., Zhong S.-L., Wu Y.-J., Xu W.-D., Xu J.-J., Tang J.-H., Zhao J.-H. (2013). Systematic expression analysis of genes related to multidrug-resistance in isogenic docetaxel- and adriamycin-resistant breast cancer cell lines. Mol. Biol. Rep..

[54] GeneCards^®^ Home Page. http://www.genecards.org/.

[55] Gene NCBI National Center for Biotechnology Information. U.S. National Library of Medicine. 8600 Rockville Pike, Bethesda, MD, USA. https://www.ncbi.nlm.nih.gov/gene.

[56] Allison K.H., Hammond M.E.H., Dowsett M., McKernin S.E., Carey L.A., Fitzgibbons P.L., Hayes D.F., Lakhani S.R., Chavez-MacGregor M., Perlmutter J. (2020). Estrogen and Progester-one Receptor Testing in Breast Cancer: ASCO/CAP Guideline Update. J. Clin. Oncol..

[57] Parker C., Castro E., Fizazi K., Heidenreich A., Ost P., Procopio G., Tombal B., Gillessen S. (2020). ESMO Clinical Practice Guidelines for diagnosis, treatment and follow-up. Ann. Oncol..

[58] Jividen K., Yang C.S., Szlachta K., Ratan A., Paschal B.M. (2018). Genomic analysis of DNA repair genes and androgen signaling in prostate cancer. BMC Cancer.

[59] Akaza H., Procopio G., Pripatnanont C., Facchini G.V., Fava S., Wheatley D., Leung K.C., Butt M., Silva A., Castillo L. (2018). Metastatic Castration-Resistant Pros-tate Cancer Previously Treated With Docetaxel-Based Chemotherapy: Treatment Patterns from the PROXIMA Prospective Registry. J. Glob. Oncol..

[60] Farha N.G., Kasi A. (2020). Docetaxel. [Updated 2020 Apr 13]. StatPearls [Internet].

[61] Yiannakopoulou E.C. (2014). Interaction of Green Tea Catechins with Breast Cancer Endocrine Treatment: A Systematic Review. Pharmacol..

[62] Wang P., Henning S.M., Heber D., Vadgama J.V. (2015). Sensitization to docetaxel in prostate cancer cells by green tea and quer-cetin. J. Nutr. Biochem..

[63] Kilic U., Sahin K., Tuzcu M., Basak N., Orhan C., Elibol-Can B., Kilic E., Sahin F., Kucuk O. (2015). Enhancement of Cisplatin sensitivity in human cervical cancer: Epigallocatechin-3-gallate. Front Nutr..

[64] Przystupski D., Michel O., Rossowska J., Kwiatkowski S., Saczko J., Kulbacka J. (2019). The modulatory effect of green tea catechin on drug resistance in human ovarian cancer cells. Med. Chem. Res..

[65] Flores-Pérez A., Marchat L.A., Sánchez L.L., Romero-Zamora D., Arechaga-Ocampo E., Ramírez-Torres N., Chávez J.D., Carlos-Reyes Á., Astudillo-de la Vega H., Ruiz-García E. (2016). Differ-ential proteomic analysis reveals that EGCG inhibits HDGF and activates apoptosis to increase the sensitivity of non-small cells lung cancer to chemotherapy. Proteom. Clin. Appl..

[66] Damianaki A., Bakogeorgou E., Kampa M., Notas G., Hatzoglou A., Panagiotou S., Gemetzi C., Kouroumalis E., Martin P.-M., Castanas E. (2000). Potent inhibitory action of red wine polyphenols on human breast cancer cells. J. Cell. Biochem..

[67] Goodin M.G., Fertuck K.C., Zacharewski T.R., Rosengren R.J., Goodin M.G., Fertuck K.C., Zacharewski T.R., Rosengren R.J. (2002). Estrogen Receptor-Mediated Actions of Polyphenolic Catechins in Vivo and in Vitro. Toxicol. Sci..

[68] Kuruto-Niwa R., Inoue S., Ogawa S., Muramatsu M., Nozawa R. (2000). Effects of tea catechins on the ERE-regulated estrogenic activity. J. Agric. Food Chem..

[69] Puranik N.V., Srivastava P., Bhatt G., John Mary D.J.S., Limaye A.M., Sivaraman J. (2019). Determination and analysis of agonist and antagonist potential of naturally occurring flavonoids for estrogen receptor (ERα) by various param-eters and molecular modelling approach. Sci. Rep..

[70] Ratna W.N., Bhatt V.D., Chaudhary K., Ariff A.B., Bavadeka S.A., Boggeti R. (2012). Selective Estrogen-Receptor Modulators Genistein, Resveratrol, and Catechin fail to stimulate the Hepatic Expression of Estrogen-Responsive Genes Encoding the Avian Apolipoprotein II and Vitellogenin. FASEB J..

[71] Hallman K., Aleck K., Quigley M., Dwyer B., Lloyd V., Szmyd M. (2017). The regulation of steroid receptors by epigallocate-chin-3-gallate in breast cancer cells. Breast Cancer.

[72] De Amicis F., Russo A., Avena P., Santoro M., Vivacqua A., Bonofiglio D., Mauro L., Aquila S., Tramontano D., Fuqua S.A. (2013). In vitro mechanism for downregulation of ER-α expression by epigallocatechin gallate in ER+/PR+ human breast cancer cells. Mol. Nutr. Food Res..

[73] Farabegoli F., Barbi C., Lambertini E., Piva R. (2007). (−)-Epigallocatechin-3-gallate downregulates estrogen receptor alpha function in MCF-7 breast carcinoma cells. Cancer Detect. Prev..

[74] Siddiqui I.A., Asim M., Hafeez B.B., Adhami V.M., Tarapore R.S., Mukhtar H. (2011). Green tea polyphenol EGCG blunts androgen re-ceptor function in prostate cancer. FASEB J..

[75] Kampa M., Theodoropoulou K., Mavromati F., Pelekanou V., Notas G., Lagoudaki E.D., Nifli A.P., Morel-Salmi C., Stathopoulos E.N., Vercauteren J. (2011). Novel oligomeric proan-thocyanidin derivatives interact with membrane androgen sites and induce regression of hormone-independent prostate cancer derivatives interact with membrane androgen sites and induce regression of hormone-independent prostate cancer. J. Pharmacol. Exp. Ther..

[76] Eng E.T., Ye J., Williams D., Phung S., Moore R., Young M.K., Gruntmanis U., Braunstein G., Chen S. (2003). Suppression of estrogen biosynthesis by procyanidin dimers in red wine and grape seeds. Cancer Res..

[77] Eng E.T., Williams D., Mandava U., Kirma N., Tekmal R.R., Chen S. (2001). Suppression of aromatase (estrogen synthetase) by red wine phytochemicals. Breast Cancer Res. Treat..

[78] Eng E.T., Williams D., Mandava U., Kirma N., Tekmal R.R., Chen S. (2006). Anti-aromatase chemicals in red wine. Ann. N. Y. Acad. Sci..

[79] Shufelt C., Merz C.N., Yang Y., Kirschner J., Polk D., Stanczyk F., Paul-Labrador M., Braunstein G.D. (2012). Red versus white wine as a nutritional aromatase inhibitor in premenopausal women: A pilot study. J. Womens Health (Larchmt).

[80] Hiipakka R.A., Zhang H.-Z., Dai W., Dai Q., Liao S. (2002). Structure–activity relationships for inhibition of human 5α-reductases by polyphenols. Biochem. Pharmacol..

[81] Choi Y.-J., Fan M., Tang Y., Yang H.P., Hwang J.-Y., Kim E.-K. (2019). In Vivo Effects of Polymerized Anthocyanin from Grape Skin on Benign Prostatic Hyperplasia. Nutrients.

[82] Choi S.Y., Ha T.Y., Ahn J.Y., Kim S.R., Kang K.S., Hwang I.K., Kim S. (2007). Estrogenic Activities of Isoflavones and Flavones and their Structure-Activity Relationships. Planta Med..

[83] Resende F.A., de Oliveira A.P., de Camargo M.S., Vilegas W., Varanda E.A. (2013). Evaluation of estrogenic potential of flavo-noids using a recombinant yeast strain and MCF7/BUS cell proliferation assay. PLoS ONE.

[84] Pan X., Zhao B., Song Z., Han S., Wang M. (2016). Estrogen receptor-α36 is involved in epigallocatechin-3-gallate induced growth inhibition of ER-negative breast cancer stem/progenitor cells. J. Pharmacol. Sci..

[85] Sweeney E.E., McDaniel R.E., Maximov P.Y., Fan P., Jordan V.C. (2012). Models and mechanisms of acquired antihormone resistance in breast cancer: Significant clinical progress despite limitations. Horm. Mol. Biol. Clin. Investig..

[86] Mota A.L., Evangelista A.F., Macedo T., Oliveira R., Scapulatempo-Neto C., Vieira R.A., Marques M.M.C. (2017). Molecular characterization of breast cancer cell lines by clinical immunohistochemical markers. Oncol. Lett..

[87] D’Amato N.C., Gordon M.A., Babbs B.L., Spoelstra N.S., Butterfield K.T.C., Torkko K.C., Phan V.T., Barton V.N., Rogers T.J., Sartorius C.A. (2016). Cooperative Dynamics of AR and ER Activity in Breast Cancer. Mol. Cancer Res..

[88] Magklara A., Brown T.J., Diamandis E.P. (2002). Characterization of androgen receptor and nuclear receptor co-regulator ex-pression in human breast cancer cell lines exhibiting differential regulation of kallikreins 2 and 3. Int. J. Cancer.

[89] Chamberlain N.L., Driver D., Miesfeld R.L. (1994). The length and location of CAG trinucleotide repeats in the androgen receptor N-terminal domain affect transactivation function. Nucleic Acids Res..

[90] Hu D.G., Hickey T., Irvine C., Wijayakumara D.D., Lu L., Tilley W.D., Selth L., MacKenzie P.I. (2014). Identification of Androgen Receptor Splice Variant Transcripts in Breast Cancer Cell Lines and Human Tissues. Horm. Cancer.

[91] Lafront C., Germain L., Weidmann C., Audet-Walsh É. (2020). A Systematic Study of the Impact of Estrogens and Selective Es-trogen Receptor Modulators on Prostate Cancer Cell Proliferation. Sci. Rep..

[92] Sasaki M., Tanaka Y., Perinchery G., Dharia A., Kotcherguina I., Fujimoto S., Dahiya R. (2002). Methylation and inactivation of estrogen, progesterone, and androgen receptors in prostate cancer. J. Natl. Cancer Inst..

[93] Sampayo R., Recouvreux S., Simian M. (2013). The hyperplastic phenotype in PR-A and PR-B transgenic mice: Lessons on the role of estrogen and progesterone receptors in the mouse mammary gland and breast cancer. Vitam. Horm..

[94] Ellem S.J., Schmitt J.F., Pedersen J.S., Frydenberg M., Risbridger G. (2004). Local Aromatase Expression in Human Prostate Is Altered in Malignancy. J. Clin. Endocrinol. Metab..

[95] Ryde C.M., Nicholls J.E., Dowsett M. (1992). Steroid and growth factor modulation of aromatase activity in MCF7 and T47D breast carcinoma cell lines. Cancer Res..

[96] Sonne-Hansen K., Lykkesfeldt A. (2005). Endogenous aromatization of testosterone results in growth stimulation of the human MCF-7 breast cancer cell line. J. Steroid Biochem. Mol. Biol..

[97] Aka J.A., Lin S.-X. (2012). Correction: Comparison of Functional Proteomic Analyses of Human Breast Cancer Cell Lines T47D and MCF7. PLoS ONE.

[98] Negri-Cesi P., Colciago A., Poletti A., Motta M. (1999). 5alpha-reductase isozymes and aromatase are differentially expressed and active in the androgen-independent human prostate cancer cell lines DU145 and PC3. Prostate.

[99] Wiebe J.P., Lewis M.J. (2003). Activity and expression of progesterone metabolizing 5alpha-reductase, 20alpha-hydroxysteroid oxi-doreductase and 3alpha(beta)-hydroxysteroid oxidoreductases in tumorigenic (MCF-7, MDA-MB-231, T-47D) and nontu-morigenic (MCF-10A) human breast cancer cells. BMC Cancer.

[100] Rice M.A., Malhotra S.V., Stoyanova T. (2019). Second-Generation Antiandrogens: From Discovery to Standard of Care in Castra-tion Resistant Prostate Cancer. Front Oncol..

[101] Kyriakopoulos C.E., Chen Y.H., Carducci M.A., Liu G., Jarrard D.F., Hahn N.M., Shevrin D.H., Dreicer R., Hussain M., Eisenberger M. (2018). Chemohormonal Therapy in Meta-static Hormone-Sensitive Prostate Cancer: Long-Term Survival Analysis of the Randomized Phase III E3805 CHAARTED Trial. J. Clin. Oncol..

[102] Lumachi F., Santeufemia D., Basso S.M. (2015). Current medical treatment of estrogen receptor-positive breast cancer. World J. Biol. Chem..

[103] Cook A., Beesley S., O’Sullivan J.M., Birtle A.J., Thalmann G., Graham J.D., Spears M.R., Brock S., Srinivasan R., Protheroe A. (2016). Addition of docetaxel, zoledronic acid, or both to first-line long-term hormone therapy in prostate cancer (STAMPEDE): Survival results from an adaptive, multiarm, multistage, platform randomised controlled trial. Lancet.

[104] Lo Y.-C., Cormier O., Liu T., Nettles K.W., Katzenellenbogen J.A., Stearns T., Altman R.B. (2019). Pocket similarity identifies selective estrogen receptor modulators as microtubule modulators at the taxane site. Nat. Commun..

[105] Mukhtar E., Adhami V.M., Mukhtar H. (2014). Targeting Microtubules by Natural Agents for Cancer Therapy. Mol. Cancer Ther..

[106] Banerjee S., Hwang D.J., Li W., Miller D.D. (2016). Current Advances of Tubulin Inhibitors in Nanoparticle Drug Delivery and Vas-cular Disruption/Angiogenesis. Molecules.

[107] Sumit H., Tehseen D., Lalit S. (2019). Molecular. Docking studies of Filarial β-Tubulin protein models with anti-filarial phytochem-icals. Biomed. Biotechnol. Res. J..

[108] Chakrabarty S., Ganguli A., Das A., Nag D., Chakrabarti G. (2015). Epigallocatechin-3-gallate shows anti-proliferative activity in HeLa cells targeting tubulin-microtubule equilibrium. Chem. Interact..

[109] Zhang Y., Xu Y.Y., Sun W.J., Zhang M.H., Zheng Y.F., Shen H.M., Yang J., Zhu X.Q. (2016). FBS or BSA Inhibits EGCG Induced Cell Death through Covalent Binding and the Reduction of Intracellular ROS Production. Biomed. Res. Int..

[110] Santamaría D., Barrière C., Cerqueira A., Hunt S., Tardy C., Newton K., Caceres J., Dubus P., Malumbres M., Barbacid M. (2007). Cdk1 is sufficient to drive the mammalian cell cycle. Nat. Cell Biol..

[111] Karimian A., Ahmadi Y., Yousefi B. (2016). Multiple functions of p21 in cell cycle, apoptosis and transcriptional regulation after DNA damage. DNA Repair.

[112] Cao J., Zhu Z., Wang H., Nichols T.C., Lui G.Y.L., Deng S., Rejto P.A., VanArsdale T., Hardwick J.S., Weinrich S.L. (2019). Combining CDK4/6 inhibition with taxanes enhances anti-tumor efficacy by sustained impairment of pRB-E2F pathways in squamous cell lung cancer. Oncogene.

[113] Ding L., Cao J., Lin W., Chen H., Xiong X., Ao H., Yu M., Lin J., Cui Q. (2020). The Roles of Cyclin-Dependent Kinases in Cell-Cycle Progression and Therapeutic Strategies in Human Breast Cancer. Int. J. Mol. Sci..

[114] Chohan T.A., Qayyum A., Rehman K., Tariq M., Akash M.S.H. (2018). An insight into the emerging role of cyclin-dependent ki-nase inhibitors as potential therapeutic agents for the treatment of advanced cancers. Biomed Pharmacother..

[115] Bai J., Li Y., Zhang G. (2017). Cell cycle regulation and anticancer drug discovery. Cancer Biol. Med..

[116] Zeng X., Xu W.K., Lok T.M., Ma H.T., Poon R.Y.C. (2019). Imbalance of the spindle-assembly checkpoint promotes spindle poi-son-mediated cytotoxicity with distinct kinetics. Cell Death Dis..

[117] Liu R., Liu C., Chen D., Yang W.H., Liu X., Liu C.G., Dugas C.M., Tang F., Zheng P., Liu Y. (2015). FOXP3 Controls an miR-146/NF-κB Negative Feedback Loop That Inhibits Apoptosis in Breast Cancer Cells. Cancer Res..

[118] Li D., Hu C., Li H. (2018). Survivin as a novel target protein for reducing the proliferation of cancer cells (Review). Biomed. Rep..

[119] Karamouzis M.V., Papavassiliou K.A., Adamopoulos C., Papavassiliou A.G. (2016). Targeting Androgen/Estrogen Receptors Crosstalk in Cancer. Trends Cancer.

[120] Chaudhri R.A., Hadadi A., Lobachev K.S., Schwartz Z., Boyan B.D. (2014). Estrogen receptor-alpha 36 mediates the an-ti-apoptotic effect of estradiol in triple negative breast cancer cells via a membrane-associated mechanism. Biochim. Biophys. Acta.

[121] Papadopoulou N., Papakonstanti E.A., Kallergi G., Alevizopoulos K., Stournaras C. (2009). Membrane androgen receptor acti-vation in prostate and breast tumor cells: Molecular signaling and clinical impact. IUBMB Life.

[122] Giovannelli P., Di Donato M., Auricchio F., Castoria G., Migliaccio A. (2019). Androgens Induce Invasiveness of Triple Negative Breast Cancer Cells through AR/Src/PI3-K Complex Assembly. Sci. Rep..

[123] Dong Y., Bai S., Zhang B.Y. (2019). Impact of taxanes on androgen receptor signaling. Asian J. Androl..

[124] McLoughlin E.C., O’Boyle N.M. (2020). Colchicine-Binding Site Inhibitors from Chemistry to Clinic: A Review. Pharmaceuticals.

[125] COSMIC, the Catalogue of Somatic Mutations in Cancer. Cell Lines Project. http://cancer.sanger.ac.uk/cancergenome/projects/cell_lines/.

[126] Núñez-Iglesias M.J., Novío S., García C., Pérez-Muñuzuri E., Soengas P., Cartea E., Velasco P., Freire-Garabal M. (2019). Glu-cosinolate-Degradation Products as Co-Adjuvant Therapy on Prostate Cancer in Vitro. Int. J. Mol. Sci..

[127] Xiao D., Choi S., Johnson D.E., Vogel V.G., Johnson C.S., Trump D.L., Lee Y.J., Singh S.V. (2004). Diallyl trisulfide-induced apoptosis in human prostate cancer cells involves c-Jun N-terminal kinase and extracellular-signal regulated kinase-mediated phosphorylation of Bcl-2. Oncogene.

[128] Mao L., Yang C., Wang J., Li W., Wen R., Chen J., Zheng J. (2013). SATB1 is overexpressed in metastatic prostate cancer and promotes prostate cancer cell growth and invasion. J. Transl. Med..

